# Development of an oligosaccharide library to characterise the structural variation in glucuronoarabinoxylan in the cell walls of vegetative tissues in grasses

**DOI:** 10.1186/s13068-019-1451-6

**Published:** 2019-05-06

**Authors:** Theodora Tryfona, Mathias Sorieul, Carolina Feijao, Katherine Stott, Denis V. Rubtsov, Nadine Anders, Paul Dupree

**Affiliations:** 1 Department of Biochemistry, University of Cambridge, Hopkins Building, The Downing Site, Tennis Court Road, Cambridge, CB2 1QW UK; 2 Department of Biochemistry, University of Cambridge, Sanger Building, 80 Tennis Court Road, Cambridge, CB2 1GA UK; 3 Present Address: Scion, 49 Sala Street, Private Bag 3020, Rotorua, 3046 New Zealand; 4 Present Address: Frontiers, WeWork, 1 Fore St, London, EC2Y 5EJ UK; 5 Present Address: ideaSpace South, Cambridge Biomedical Campus, Bay 13 Hills Road, Cambridge, CB2 0SP UK

**Keywords:** Grass xylan, Bioenergy, Tissue variation, Species variation, Xylan branching, DASH

## Abstract

**Background:**

Grass glucuronoarabinoxylan (GAX) substitutions can inhibit enzymatic degradation and are involved in the interaction of xylan with cell wall cellulose and lignin, factors which contribute to the recalcitrance of biomass to saccharification. Therefore, identification of xylan characteristics central to biomass biorefining improvement is essential. However, the task of assessing biomass quality is complicated and is often hindered by the lack of a reference for a given crop.

**Results:**

In this study, we created a reference library, expressed in glucose units, of *Miscanthus sinensis* GAX stem and leaf oligosaccharides, using DNA sequencer-Assisted Saccharide analysis in high throughput (DASH), supported by liquid chromatography (LC), nuclear magnetic resonance (NMR) spectroscopy and mass spectrometry (MS). Our analysis of a number of grass species highlighted variations in substitution type and frequency of stem and leaf GAX. In miscanthus, for example, the β-Xyl*p*-(1 → 2)-α-Ara*f*-(1 → 3) side chain is more abundant in leaf than stem.

**Conclusions:**

The reference library allows fast identification and comparison of GAX structures from different plants and tissues. Ultimately, this reference library can be used in directing biomass selection and improving biorefining.

**Electronic supplementary material:**

The online version of this article (10.1186/s13068-019-1451-6) contains supplementary material, which is available to authorized users.

## Background

Plant xylan polysaccharides have attracted attention due to their numerous applications not only in the papermaking, baking and food industries but also in respect to bioenergy production. Branched xylan is the main hemicellulose in many crops. This xylan is made of a linear chain of β-(1 → 4)-linked xylopyranosyl (Xyl*p*) residues, which can be substituted by α-(1 → 2)-linked (4-*O*-methyl-)glucuronic acid ([Me]GlcA) and acetylation at the *O*-2 and/or *O*-3 positions [1]. In monocots, such as grasses, and also in gymnosperms, xylan is additionally modified by α-(1 → 3)-linked l-arabinofuranosyl residues (Ara*f*). The Ara*f* residues may be further substituted at *O*-2 with an Ara*f* or a Xyl*p* residue [2]. Cereal grain endosperm contains neutral arabinoxylan (AX), which is monosubstituted with α-(1 → 3)-linked Ara*f* residues or di-substituted with α-(1 → 2)-linked and α-(1 → 3)-linked Ara*f* residues [1, 3]. Ara*f* residues of both glucuronoarabinoxylan (GAX) and endosperm AX may be esterified with a feruloyl (Fer)- or coumaryl group at *O*-5 position [4, 5]. Feruloylation has been implicated in cross-linking of different xylan chains and in cross-linking to lignin [6, 7]. Feruloylated Ara*f* structures can be further substituted with β-(1 → 2)-linked Xyl*p* groups or additional sugars [8, 9]. Corn bran xylan was found to contain α-(1 → 2)-linked l-Gal*p* on the Xyl*p* residue of the Fer-Ara*f*-Xyl*p* oligomeric structure [10–12], and this was recently found to be present in other cereal grains as well [13, 14]. Among the ferulate-containing xylan side-chain variants, 5-O-Fer-Ara*f* structure appears most abundant, followed by the Xyl*p*-[5-O-Fer]-Ara*f* structure [8]. Grass GAX and AX is acetylated but to a lesser extent than dicot glucuronoxylan. However, in addition to acetyl groups being added to the backbone Xyl*p* residues, the Ara*f* substituents can carry acetyl groups at *O*-2 [15].

Most of the energy in the lignocellulosic biomass is locked within the secondary cell walls in the form of cellulose and xylan, which form a dense matrix with lignin [16]. Lignocellulosic plant cell wall recalcitrance is a barrier to cost-effective cellulosic biofuel production. Cell wall recalcitrance in regards to xylan is influenced by various factors: heavy substitution of xylan, which can impair the action of hydrolytic enzymes; specific branching points, which can serve as cross-linking sites with lignin and the pattern of branching, which can affect the interaction of xylan with cellulose [17]. The importance of xylan in recalcitrance is illustrated by the finding that removal of xylan in switchgrass resulted in materials that achieved nearly 100% glucose yields in subsequent enzymatic hydrolysis [18].

In order to improve the biomass of bioenergy crops and optimise the biorefining processes, information on the structure of xylan, and its variation, is crucial. However, little is known about the variability of xylan structure of grasses in, e.g. different tissues like stems and leaves. In addition, analysis methods of xylooligosaccharides have to be fast, accurate and robust. The majority of detailed structural information on xylan in grasses is based on analysis of cereal grain xylans such as corn cob, oat spelt, barley husks or wheat endosperm often using LC, NMR and MS [19–23]. More recently, structures of xylan of lignified tissues were analysed in grasses [24–26]. Oligosaccharides with different degrees of polymerisation (DP), glycosidic linkages and saccharide composition can be resolved with DNA sequencer-Assisted Saccharide analysis High throughput (DASH) which can analyse simultaneously 96 samples by capillary electrophoresis [27]. Our study provides the detailed structural characterisation of xylan oligosaccharides from *Miscanthus sinensis* stem cell walls hydrolysed with xylan-specific glycosyl hydrolases (GH) from the families 10 and 11. This information was used to generate a DASH reference library of GAX oligosaccharides with their corresponding glucose units (GU) as mobility standards. This library allows the fast and quantitative comparison of GAX oligosaccharide structures using the high-throughput method DASH, as shown by the comparison of oligosaccharide profiles of leaf and grass GAX from different grasses. Relative quantification of side chains can be achieved as exemplified by the more detailed analysis of miscanthus stem and leaf GAX.

## Results

### Development of a GAX oligosaccharide library from *Miscanthus sinensis* stem

In order to use DASH as a high-throughput method to characterise GAX structures and achieve relative quantification of different xylan substitutions, a GAX oligosaccharide library had to be created to serve as a standard for structural analysis. To develop this library, *Miscanthus sinensis* stem GAX was hydrolysed with endo-β-xylanases GH10 and GH11, respectively. Most reported xylanases classify into these two families, which are described to have slightly different substrate specificities. Briefly, GH10 xylanases are more capable of hydrolysing adjacent to substitutions of the xylan backbone, while GH11 xylanases preferably act on relatively unsubstituted parts of xylan [28, 29].

To describe the various hydrolysis products we use the heteroxylan naming system suggested by Faure et al. [30] with the exception of xylooligosaccharide standards which are described as X_1_–X_6_ corresponding to xylose, xylobiose, xylotriose, xylotetraose, xylopentaose and xylohexaose.

The DASH capillary electropherograms were first aligned using the internal mobility migration markers mixed with each sample to eliminate variation between capillaries [27]. The GAX oligosaccharide library was compiled by assigning the characterised oligosaccharide structures to DASH peaks based on their migration in DASH. The migration of the oligosaccharides was compared to the migration of dextran standards to provide migration information in a method we adapted here from liquid chromatography [31]. By providing the migration of the xylooligosaccharides in GU, the identification is more robust to any changes in capillary sequencer variation. Comparison of oligosaccharide GU migration therefore allows the fast and reliable annotation of GAX structures in unknown samples to all DASH users.

To characterise the detailed structure of GAX oligosaccharides, we separated the hydrolysis products by SEC and all fractions were analysed by DASH. Based on the result of the DASH analysis, SEC fractions were selected in which the respective oligosaccharides were highly abundant. Part of these fractions was subjected to secondary enzymatic hydrolysis followed by DASH analysis. The other part was used to separate possible structural isomers by Hydrophilic Interactions Liquid Chromatography (HILIC) followed by off-line Matrix-Assisted laser Desorption Ionisation (MALDI)-Mass Spectrometry. The oligosaccharides were then subjected to high-energy MALDI Collision-Induced Dissociation (CID) for detailed structural analysis. Ultimately, DASH peaks were matched to oligosaccharide structures characterised with HILIC–MALDI–MS/MS CID by combining data on peak abundance and enzyme sensitivity from different SEC fractions.

Based on the previously characterised composition of grass GAX [1], in the MALDI-CID spectra we assign uronic acid substitutions as GlcA and furanosyl pentosyl substitutions as Ara*f* on a 1,4-linked Xyl*p* backbone. Given that no GlcA residues on xylan have been reported to carry a methyl modification on the *O*-6, we assign methyl group modifications at *O*-4. In addition, all oligosaccharides characterised here are generated by GH10 or GH11 endo-β-1,4 xylanases and therefore the non-reducing end backbone Xyl*p* residues cannot be modified at the *O*-4. Glycosidic bond and cross-ring product ions are labelled according to the nomenclature of Domon and Costello [32]. The D, E, G and V ions are labelled according to previously established nomenclature [33–35]. We used sequential digests of the oligosaccharides with enzymes to gain further insights into the structure and to confirm the results of the MALDI-CID: arabinofuranosidases GH51 and GH62 hydrolyse single Ara*f* substitutions but not Xyl*p* [36], which helps to distinguish the nature of pentosyl side chains; glucuronidases GH67 and GH115 both remove [Me]GlcA substitutions. However, GH67 can only remove terminal GlcA from the non-reducing end, whereas GH115 preferably acts on substitutions of internal regions although it will also cleave GlcA from non-reducing terminal Xyl*p* residues albeit at a slower rate [37, 38].

**Fig. 1:**
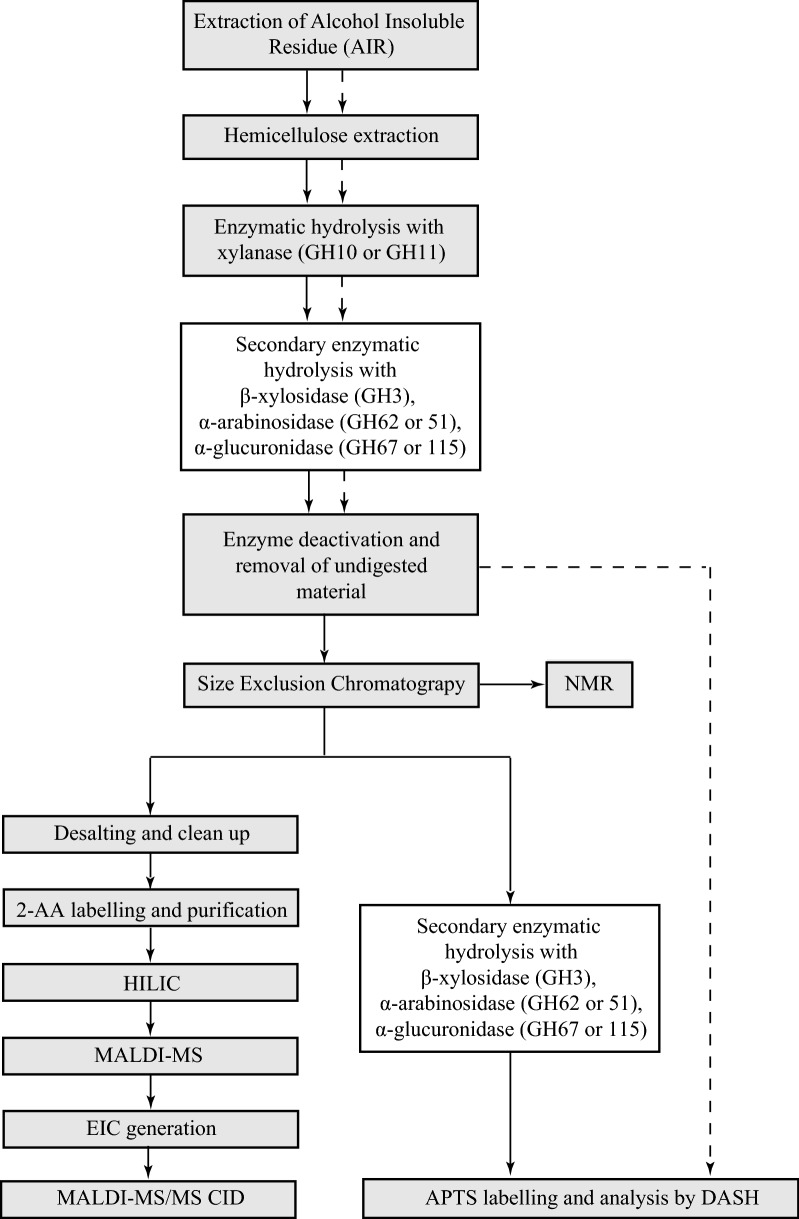
Overview of the technical procedure used to characterise GAX structures. Creation of the GAX oligosaccharide library from *Miscanthus sinensis* including extensive structural analysis (solid lines), generation of standard DASH profiles prior to SEC (dashed lines). White filled boxes indicate optional steps

An overview of the analysis is depicted in Fig. 1.

### GAX oligosaccharide profiles of xylanase GH10 and GH11 hydrolysis of *Miscanthus sinensis*

**Fig. 2:**
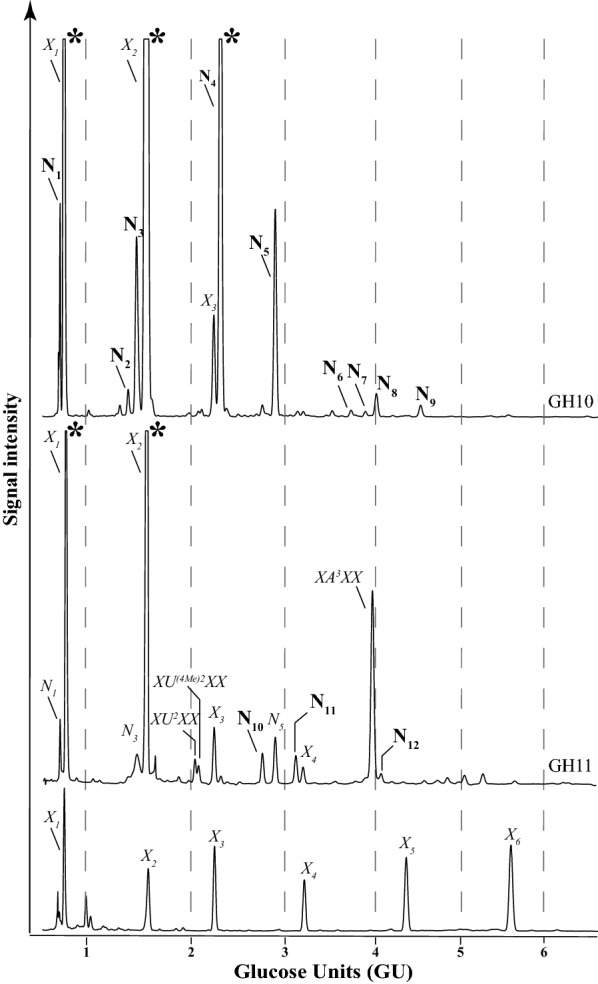
DASH profile of GAX oligosaccharides after hydrolysis with xylanases prior to SEC separation. GH10 (top panel), GH11 (middle panel), β-(1 → 4)-xylooligosaccharide standards X_1_-X_6_ (bottom panel). In addition, xylooligosaccharides XU^2^XX, XU^(4Me)2^XX and XA^3^XX as well as unknown peaks N_1_-N_12_ are labelled. Oligosaccharide migration is expressed in glucose units (GU). Asterisks (*) mark off-scale peaks

In brief, a standard DASH profile was generated by APTS labelling of GH10 and GH11 hydrolysis products of *Miscanthus sinensis* stem alcohol-insoluble residue (AIR), respectively, and analysis by DASH (Fig. 2).

**Fig. 3:**
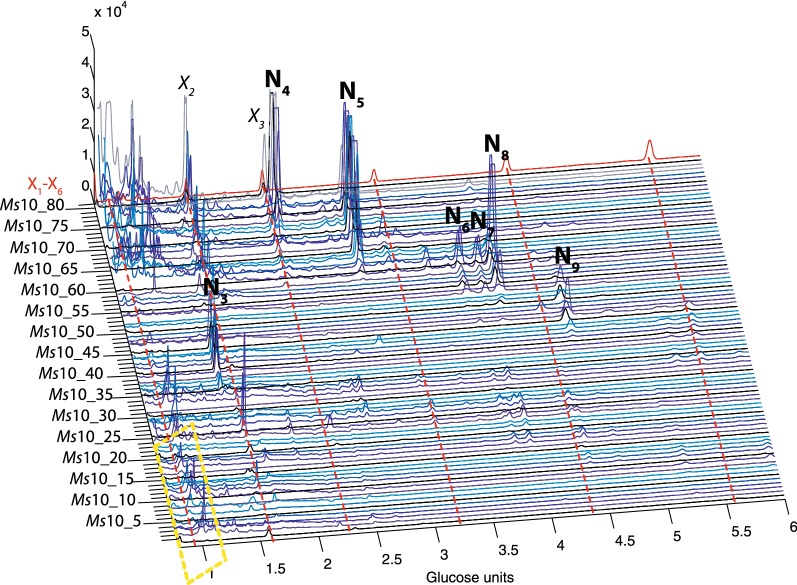
DASH profile of SEC fractions from GH10 hydrolysis of miscanthus stem. 80 SEC fractions were separated and analysed by DASH (blue and grey traces), xylooligosaccharide standards X_1_–X_6_ (red trace). Area marked with yellow dashed box corresponds to background noise. Unknown peaks N_3_–N_9_ are labelled

In order to partially separate the oligosaccharides for further structural analysis, the hydrolysis products were subjected to Size Exclusion Chromatography (SEC). Eighty *Miscanthus sinensis* GH10 and GH11 oligosaccharide SEC fractions (*Ms*10_1–80 and *Ms*11_1–80) were collected and labelled with APTS and analysed by DASH, revealing separation of a number of oligosaccharides by SEC (Fig. 3 and Additional file 1: Figure S1) in comparison to the standard DASH profile prior to SEC (Fig. 2). Figure 3 and Additional file 1: Figure S1 also show the power of DASH to study a large number of oligosaccharide mixtures, to aid interpretation of the SEC separation and selection of fractions of interest.

Some oligosaccharides separated by DASH eluted at the same time as β-(1 → 4)-xylooligosaccharide (X_1_–X_6_) standards; other oligosaccharides (namely XU^2^XX, XU^(4Me)2^XX and XA^3^XX) were previously structurally characterised [27–29]. The identity of their DASH peaks was confirmed by MALDI-CID (data not shown). Remaining unknown peaks were labelled N_1–12_ (Fig. 2). The detailed results of the structural analysis of each oligosaccharide are described below. Table 1 summarises the GAX oligosaccharide structures generated by GH10 and GH11 hydrolysis and their corresponding GU. Table 2 summarises the sensitivity/resistance to enzymatic hydrolysis of GAX oligosaccharides and Additional file 1: Figure S2 shows the sensitivity/resistance of selected oligosaccharides to enzymatic hydrolysis.

**Table 1:** Structures of GAX oligosaccharides and their migration positions by DASH expressed in glucose units (GU)

Nomenclature	Structure	Unknown structure	MALDI-CID or NMR figure	Glucose units
U^(4Me)2^X	4-*O*-Me-α-Glc*p*A-(1 → 2)-β-Xyl*p*-(1 → 4)-Xyl*p*	N_1_	S3	0.72
X	Xyl*p*			0.80
U^2^XX	α-Glc*p*A-(1 → 2)-β-Xyl*p*-(1 → 4)-β-Xyl*p*-(1 → 4)-Xyl*p*	N_2_		1.40
U^(4Me)2^XX	4-*O*-Me-α-Glc*p*A-(1 → 2)-β-Xyl*p*-(1 → 4)-β-Xyl*p*-(1 → 4)-Xyl*p*	N_3_	S4	1.48
X_2_	β-Xyl*p*-(1 → 4)-Xyl*p*			1.57
*XU* ^*2*^ *XX*	β-Xyl*p*-(1 → 4)-[α-Glc*p*A-(1 → 2)]-β-Xyl*p*-(1 → 4)-β-Xyl*p*-(1 → 4)-Xyl*p*			2.03
*XU* ^*(4Me)2*^ *XX*	β-Xyl*p*-(1 → 4)-[4-*O*-Me-α-Glc*p*A-(1 → 2)]-β-Xyl*p*-(1 → 4)-β-Xyl*p*-(1 → 4)-Xyl*p*			2.07
X_3_	β-Xyl*p*-(1 → 4)-β-Xyl*p*-(1 → 4)-Xyl*p*			2.24
A^3^X	α-Ara*f*-(1 → 3)-β-Xyl*p*-(1 → 4)-Xyl*p*	N_4_	S5	2.31
A^3^U^(4Me)2^XX	α-Ara*f*-(1 → 3)-β-Xyl*p*-(1 → 4)-[4-*O*-Me-α-Glc*p*A-(1 → 2)]-β-Xyl*p*-(1 → 4)-β-Xyl*p*-(1 → 4)-Xyl*p*	N_10_	S8	2.74
XA^3^X	β-Xyl*p*-(1 → 4)-[α-Ara*f*-(1 → 3)]-β-Xyl*p*-(1 → 4)-Xyl*p*	N_5_	S6	2.87
B^2,3^U^(4Me)2^XX or D^2,3^U^(4Me)2^XX	Ara*f*-(1 → 2)-α-Ara*f*-(1 → 3)-β-Xyl*p*-(1 → 4)-[α-Glc*p*A-(1 → 2)]-β-Xyl*p*-(1 → 4)-β-Xyl*p*-(1 → 4)-Xyl*p* β-Xyl*p*-(1 → 2)-α-Ara*f*-(1 → 3)-β-Xyl*p*-(1 → 4)-[α-Glc*p*A-(1 → 2)]-β-Xyl*p*-(1 → 4)-β-Xyl*p*-(1 → 4)-Xyl*p*	N_11_	S9	3.09
D^2,3^X	β-Xyl*p*-(1 → 2)-α-Ara*f*-(1 → 3)-β-Xyl*p*-(1 → 4)-Xyl*p*	M_1_	S12	3.12
X_4_	β-Xyl*p*-(1 → 4)-β-Xyl*p*-(1 → 4)-β-Xyl*p*-(1 → 4)-Xyl*p*			3.17
A^3^A^3^X	α-Ara*f*-(1 → 3)-β-Xyl*p*-(1 → 4)-[α-Ara*f*-(1 → 3)]-β-Xyl*p*-(1 → 4)-Xyl*p*	N_6_ (Z_1_)	5A	3.70
A^3^XXX	α-Ara*f*-(1 → 3)-β-Xyl*p*-(1 → 4)-β-Xyl*p*-(1 → 4)-β-Xyl*p*-(1 → 4)-Xyl*p*	N_7_ (Z_2_)	5B	3.85
*XA* ^*3*^ *XX*	β-Xyl*p*-(1 → 4)-[α-Ara*f*-(1 → 3)]-β-Xyl*p*-(1 → 4)-β-Xyl*p*-(1 → 4)-Xyl*p*			3.92
D^2,3^XX	β-Xyl*p*-(1 → 2)-α-Ara*f*-(1 → 3)-β-Xyl*p*-(1 → 4)-β-Xyl*p*-(1 → 4)-Xyl*p*	N_8_ (Z_3_)	5C 5D	3.97
XA^3^XU^(4Me)2^XX	β-Xyl*p*-(1 → 4)-[α-Ara*f*-(1 → 3)]-β-Xyl*p*-(1 → 4)-β-Xyl*p*-(1 → 4)-[4-*O*-Me-α-Glc*p*A-(1 → 2)]-β-Xyl*p*-(1 → 4)-β-Xyl*p*-(1 → 4)-Xyl*p*	N_12_	S10	4.01
X_5_	β-Xyl*p*-(1 → 4)-β-Xyl*p*-(1 → 4)-β-Xyl*p*-(1 → 4)-β-Xyl*p*-(1 → 4)-Xyl*p*			4.32
XA^3^A^3^X	β-Xyl*p*-(1 → 4)-[α-Ara*f*-(1 → 3)]-β-Xyl*p*-(1 → 4)-[α-Ara*f*-(1 → 3)]-β-Xyl*p*-(1 → 4)-Xyl*p*	N_9_	S7	4.47
XD^2,3^XX	β-Xyl*p*-(1 → 4)-[β-Xyl*p*-(1 → 2)-α-Ara*f*-(1 → 3)]-β-Xyl*p*-(1 → 4)-β-Xyl*p*-(1 → 4)-Xyl*p*	M_2_	S11	4.83
X_6_	β-Xyl*p*-(1 → 4)-β-Xyl*p*-(1 → 4)-β-Xyl*p*-(1 → 4)-β-Xyl*p*-(1 → 4)-β-Xyl*p*-(1 → 4)-Xyl*p*			5.54

**Table 2:** Enzymatic analysis of GAX oligosaccharides

Oligosaccharide	Unknown structure	GH10 product	GH11 product	Enzyme sensitivity				
				*Cg*GH3 xylosidase	*Pc*GH51 α-arabinosidase	*Pa*GH62 α-arabinosidase	*Cj*GH67 α-glucuronidase	*Bo*GH115 α-glucuronidase
U^(4Me)2^X	N_1_	●●	●	−	−	−	−	+
U^2^XX	N_2_	●		−	−	−	+	+
U^(4Me)2^XX	N_3_	●●	●	−	−	−	+	+
A^3^X	N_4_	●●		−	+	+	NT	−
*XU* ^*2*^ *XX*	−		●	−	−	−	+	+
*XU* ^*(4Me)2*^ *XX*	−		●	−	−	−	+	+
XA^3^X	N_5_	●●	●●	NT	+	+	NT	−
A^3^A^3^X	N_6_ (Z_1_)	●		NT	+	−	NT	−
A^3^XXX	N_7_ (Z_2_)	●		NT	+	+	NT	NT
*XA* ^*3*^ *XX*	−		●●	−	NT	+	NT	−
D^2,3^XX	N_8_ (Z_3_)	●		+	−	−	NT	−
XA^3^A^3^X	N_9_	●		−	+	−	NT	NT
A^3^U^(4Me)2^XX	N_10_		●	NT	+	−	NT	−
B^2,3^U^(4Me)2^XX or D^2,3^U^(4Me)2^XX	N_11_		●	−	−	NT	NT	−
XA^3^XU^(4Me)2^XX	N_12_		●	NT	+	+	NT	+
D^2,3^X	M_1_	●		+	−	−	NT	−
XD^2,3^XX	M_2_		●	+	−	−	NT	−

The dots indicate, which xylanase produces the oligosaccharide; one dot (●) indicates minor products while two dots (●●) indicate major products. Oligosaccharides sensitive to GH enzymes are marked with a plus (+), those resistant with a minus (−); *NT* not tested. Oligosaccharides XU^2^XX, X^(4Me)2^XX and XA^3^XX have been previously structurally characterised [27–29]

### Structural characterisation of the oligosaccharides N_1_–N_9_ from the xylanase GH10 digest of *Miscanthus sinensis*

After GH10 hydrolysis, three oligosaccharides co-eluted with X_1_, X_2_ and X_3_ of the xylooligosaccharide standards in DASH. We identified nine additional peaks from oligosaccharides of unknown structure which were named N_1_–N_9_ (Fig. 2). The [Me]GlcA modified hydrolysis products mainly accumulated in earlier SEC fractions independent of their molecular size (Fig. 3, fractions *Ms*10_24–46) because of the negative charge of the Bio-Gel P2, which results in the exclusion of uronic acids [39].

The oligosaccharide N_1_ in the DASH trace was analysed from the unfractionated GH10 miscanthus stem hydrolysis products (oligosaccharide mix prior to SEC fractionation). The [M + Na]^+^ ion at *m/z* 616.0 corresponds to a Pent_2_ structure modified with one MeGlcA. The MALDI-CID reveals the structure of N_1_ as U^(4Me)2^X (Additional file 1: Figure S3).

The Y_2_ ion (*m/z* 426.0) shows there are two Xyl*p* residues in the backbone. The presence of the significant Y_1_ ion (*m/z* 294.1) indicates that the reducing-end Xyl*p* is unsubstituted. The ^0,2^X_1_ ion (*m/z* 523.9) indicates that the non-reducing-end Xyl*p* is modified at the *O*-2 with a MeGlcA, while the presence of the V_3_ product ion (*m/z* 539.9) indicates that the GlcA is modified with a methyl group at the *O*-4. Consistent with this, N_1_ oligosaccharide was sensitive to GH115 glucuronidase digestion, although resistant to GH67 hydrolysis (Table 2 and Additional file 1: Figure S2A) due to it not being an ideal substrate for the GH67 enzyme [37].

The oligosaccharide N_2_ was in low abundance in the DASH trace and MALDI-CID showed it is the unmethylated counterpart of the N_3_ oligosaccharide (data not shown).

The oligosaccharide N_3_ in the DASH trace was analysed from fraction *Ms*10_40. The [M + Na]^+^ ion at *m/z* 748.1 corresponds to a Pent_3_ structure modified with one MeGlcA. The MALDI-CID reveals the structure of N_3_ as U^(4Me)2^XX (Additional file 1: Figure S4): The series of Y and cross-ring ^1,5^X ions gives crucial sequence information showing the positioning of the [Me]GlcA on the xylan backbone. The cross-ring ^0,2^X_2_ ion (*m/z* 655.9) indicates that the non-reducing-end Xyl*p* is modified at the *O*-2 with a methylated uronic acid. The non-reducing end E_2_ (*m/z* 461.0) product ion confirms this linkage assignment and the presence of V_4_ (*m/z* 671.9) product ion indicates that the GlcA is modified with a methyl group at the *O*-4. The N_3_ oligosaccharide was susceptible to digestion with GH67 and GH115 glucuronidase (Table 2 and Additional file 1: Figure S2A), with the GH67-sensitivity confirming that the GlcA substitution is at the non-reducing end.

The oligosaccharide N_4_ in the DASH trace was analysed from fraction *Ms*10_75. The [M + Na]^+^ ion at *m/z* 558.1 corresponds to a Pent_3_ structure, but it does not co-migrate with Xyl_3_ in DASH, suggesting it is a substituted Xyl_2_. The MALDI-CID reveals the structure of N_4_ as A^3^X (Additional file 1: Figure S5): A major Y_1_ ion (*m/z* 294.1) is indicative of an unsubstituted reducing-end Xyl*p*. The presence of the ^0,2^X_1_ ion (*m/z* 336.1) and the E_2_ product ion (*m/z* 271.1) indicates that the second Xyl*p* from the reducing-end is unsubstituted at the *O*-2. The presence of the G_3_ ion (*m/z* 510.7) indicates the pentose substitution is Ara*f*. Consistent with this, N_4_ oligosaccharide was sensitive to GH62 and GH51 arabinosidase digestions (Table 2).

The oligosaccharide N_5_ in the DASH trace was analysed from fraction *Ms*10_65. The [M + Na]^+^ ion at *m/z* 690.1 corresponds to a Pent_4_ structure, but it does not co-migrate with Xyl_4_ in DASH suggesting it is a substituted Xyl_3_. The MALDI-CID reveals the structure of N_5_ as XA^3^X (Additional file 1: Figure S6).

The ^1,5^X ion shows that the 2-AA-derivatised reducing-end Xyl*p* is not modified. The cross-ring ^0,2^X_1_ ion (*m/z* 336.1) indicates that the *O*-2 in the middle Xyl*p* is also not substituted. This is supported by the presence of the E_2_ (*m/z* 403.1) product ion, while the presence of the W_2_ sugar lactone ion (*m/z* 424.1) [29] indicates that the middle Xyl*p* is substituted at *O*-3 with a pentose residue. The presence of the G_3α_ ion (*m/z* 642.0) indicates the substitution is Ara*f*. Consistent with this, the N_5_ oligosaccharide was sensitive to GH62 and GH51 arabinosidase digestions (Table 2).

**Fig. 4:**
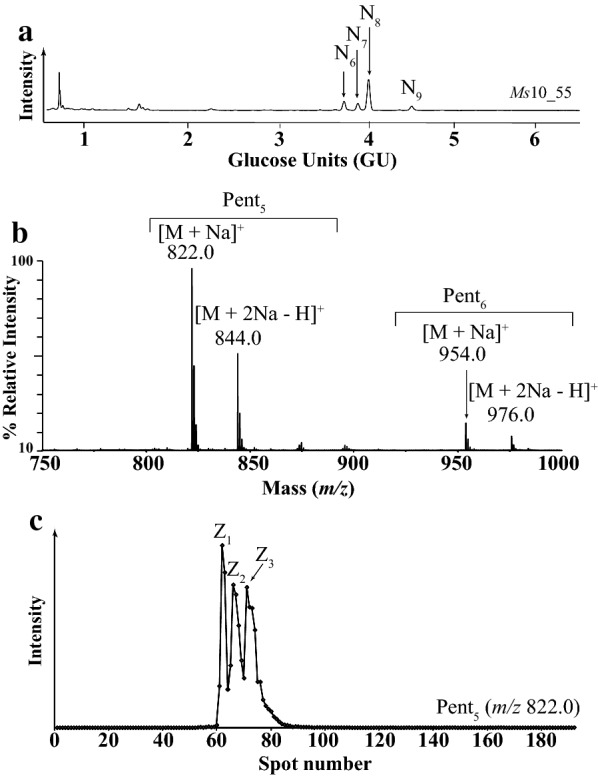
Characterisation of oligosaccharides in SEC fraction *Ms*10_55. **a** DASH capillary electropherograms showing four unknown oligosaccharides N_6_, N_7_, N_8_ and N_9_. **b** MALDI-ToF–MS of the 2-AA labelled oligosaccharides, showing a major sodiated and doubly sodiated ion corresponding to Pent_5_ (*m/z* 822.0, *m/z* 844.0, respectively) and a minor ion corresponding to Pent_6_ (*m/z* 954.0 and 976.0, respectively). **c** HILIC separation of structural isomers of *m/z* 822.0 [M + Na]^+^ followed by off-line-MALDI-ToF–MS, results in the EIC showing three structural isomers Z_1_, Z_2_ and Z_3_, respectively

The oligosaccharides N_6_–N_8_ in the DASH trace were analysed from fraction *Ms*10_55 (Fig. 4a, the structure N_9_ was more abundant in fraction *Ms*10_45 and corresponds to a Pent_6_ oligosaccharide). N_6_–N_8_ oligosaccharides had the same *m/z* 822.0 [M + Na]^+^ corresponding to Pent_5_ structural isomers (Fig. 4b). The extracted ion chromatogram (EIC) from off-line HILIC-MALDI-CID mass spectrometry of 2-AA labelled SEC fractions showed that *Ms*10_55, contained three structural isomers of Pent_5_ observed as [M + Na]^+^ at *m/z* 822.0 (Z_1_–Z_3_, Fig. 4c).

Arabinofuranosidase digestion of the oligosaccharides followed by DASH and HILIC-MALDI showed that the oligosaccharides Z_1_, Z_2_ and Z_3_ correspond to N_6_, N_7_ and N_8_ (Table 2).

**Fig. 5:**
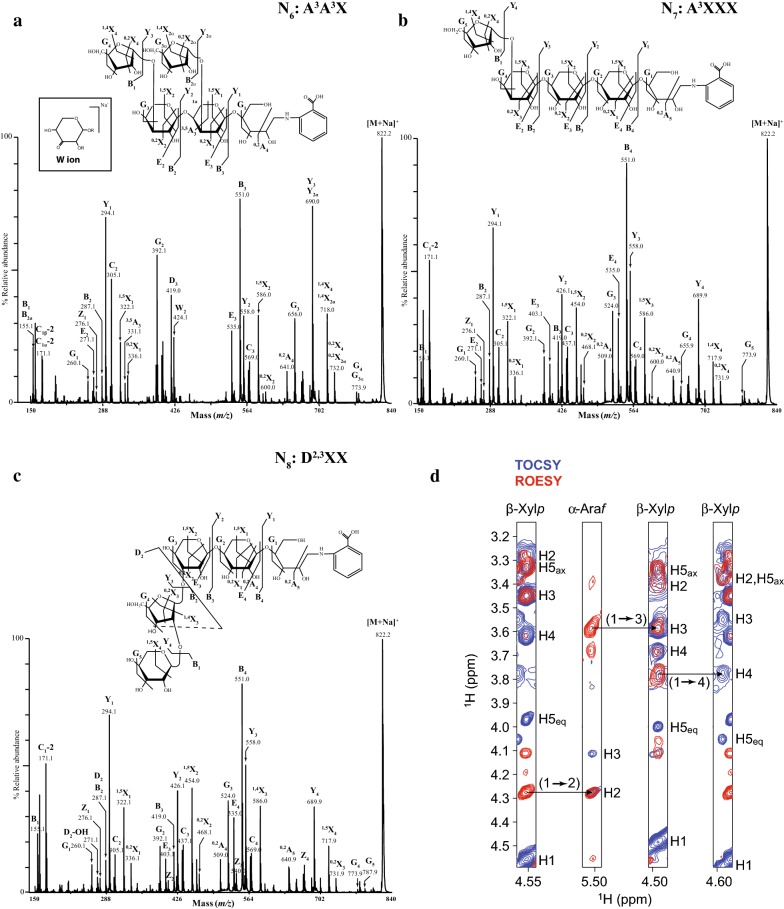
High-energy MALDI-CID spectra of oligosaccharides in SEC fraction *Ms*10_55. **a** Z_1_ structure (N_6_ in DASH) A^3^A^3^X. Inset: proposed chemical structure for W product ion [29]; **b** Z_2_ structure (N_7_ in DASH) A^3^XXX; **c** Z_3_ structure (N_8_ in DASH) D^2,3^XX. **d** NMR analysis of the Z_3_ structure. H-1 strip plots from 2D ^1^H-^1^H TOCSY (blue) and ROESY (red) spectra showing the NOE connectivity arising from the β-Xyl*p*-(1 → 2)-α-Ara*f*-(1 → 3)-β-Xyl*p*-(1 → 4)-β-Xyl*p* glycosidic linkages

The MS/MS spectra for N_6_, N_7_ and N_8_ oligosaccharides are shown in Fig. 5.

The presence of the G ions (*m/z* 773.9) in the N_6_, N_7_ and N_8_ spectra indicates Ara*f* substitution. The presence of the ^1,5^X_1_ cross-ring ion showed that the 2-AA labelled reducing-end Xyl*p* is not substituted in any of the three structural isomers. The significant Y_2_, ^1,5^X_2_ and ^0,2^X_2_ ions (*m/z* 426.1, 454.0 and 468.1, respectively) in the spectra of N_7_ and N_8_ indicate that the second Xyl*p* residue from the reducing end is also not substituted. In contrast, the absence of these ions from the N_6_ spectrum and the concomitant presence of the W_2_ (*m/z* 424.1) sugar lactone ion indicate that in this isomer the second Xyl*p* residue from the reducing-end is substituted at *O*-3. The presence of the G_3_ product ion at *m/z* 656.0 in the spectrum of N_6_ indicates that the third Xyl*p* residue from the reducing end is also substituted at the *O*-3 with Ara*f*. This is confirmed by the presence of the E_2_ (*m/z* 271.1) product ion which indicates that the third Xyl*p* from the reducing end is not substituted at *O*-2. Hence the N_6_ structure was identified as A^3^A^3^X. Consistent with this, the N_6_ oligosaccharide was sensitive to GH51 arabinosidase digestion. Possibly, due to steric hindrance of Ara*f* substitutions on two consecutive Xyl*p* residues, N_6_ was resistant to GH62 hydrolysis (Table 2).

The presence of the series of B, Y and ^1,5^X ions differing by 132 Da in the MALDI-CID spectra of the N_7_ and N_8_ structural isomers, indicates the linear sequence of pentoses. Based on the MALDI-CID spectra alone the precise structure of N_7_ and N_8_ oligosaccharides could not be identified. However, N_7_ was found to be sensitive to both GH62 and GH51 arabinofuranosidases, while N_8_ was resistant to both enzymes. Taking the MALDI-CID data and the information from enzyme sensitivity together, N_7_ was identified as A^3^XXX. N_8_ was further analysed by Nuclear Magnetic Resonance (NMR) to gain further insight into the detailed structure of this oligosaccharide (Fig. 5d and Additional file 2: Table S1).

Chemical-shift assignments were obtained using 2D ^1^H-^1^H TOCSY (TOtal Correlated SpectroscopY) and ROESY (Rotating-frame Overhauser Effect SpectroscopY) alongside 2D ^13^C HSQC (Heteronuclear Single Quantum Correlation) and HSQC-TOCSY experiments. The non-reducing-end Xyl*p* residue was readily identified and the chemical shifts of the H-1 and C-1 were consistent with a β configuration. The Xyl*p* (1 → 2) linkage to α-Ara*f* was apparent from the intense NOE from Xyl*p* H-1 to Ara*f* H-2 taken together with the downfield shift of Ara*f* C-2 characteristic of a glycosidic bond. The Ara*f*-(1 → 3)-Xyl*p* and Xyl*p*-(1 → 4)-Xyl*p* links were also confirmed by a combination of NOEs and the downfield ^13^C shifts of the linked carbon. H3 and H4 assignments of β-Xyl*p* were apparent from relative TOCSY and NOESY intensities of the cross-peak connecting to H1 (both were stronger for H3). The reducing-end Xyl*p* could not be identified due to peak broadening and overlap (the peaks for the reducing-end-adjacent Xyl*p* are also significantly broadened). Our chemical-shift assignments are in accordance with previous ^1^H and ^13^C NMR analysis of a feruloylated D^2,3^X oligosaccharide from shoots of wiregrass (*Cynodon dactylon*) [40], except the nuclei involved in the feruloyl linkage. Therefore, structure N_8_ was identified as D^2,3^XX. In addition, this structure was found to be sensitive to a GH3 β-xylosidase (*Cg*GH3) that cleaves terminal Xyl*p* residues from β-Xyl*p*-(1 → 2)-Ara*f*-(1 → 3)-Xyl*p* structures, confirming the assignment (Table 2).

The oligosaccharide N_9_ in the DASH trace was analysed from fraction *Ms*10_45. The [M + Na]^+^ ion at *m/z* 954.3 corresponds to a Pent_6_ structure. The MALDI-CID reveals the structure of N_9_ as XA^3^A^3^X (Additional file 1: Figure S7).

The ^1,5^X_1_ (*m/z* 322.1) and the Y_1_ (*m/z* 294.2) ions show that the reducing-end Xyl*p* residue is unsubstituted. The reducing-end G_2_ (*m/z* 392.2) and G_3_ (*m/z* 656.1) product ions show that the second and third Xyl*p* residues from the reducing end are substituted at *O*-3. The presence of the G3_α_/G4_β_ ion (*m/z* 906.4, loss of 48 Da) indicate the presence of at least one terminal Ara*f*. GH51 arabinosidase hydrolysis resulted in an oligosaccharide co-migrating with xylotetraose (X_4_; data not shown), confirming that both pentose substitutions are Ara*f* residues. Similar to N_6_ oligosaccharide, N_9_ was resistant to GH62 arabinosidase hydrolysis (Table 2 and Additional file 1: Figure S2C).

### Structural characterisation of the oligosaccharides N_10_, N_11_ and N_12_ from the xylanase GH11 digest of *Miscanthus sinensis*

After GH11 hydrolysis, four oligosaccharides co-eluted with X_1_, X_2_, X_3_ and X_4_ of the xylooligosaccharide standards; three oligosaccharides were structurally characterised with high-energy MALDI-CID and were identical to the previously characterised xylooligosaccharides: XU^2^XX and XU^(4Me)2^XX and XA^3^XX. Furthermore, three oligosaccharides, N_1_, N_3_ and N_5_, were identified based on their GU unit assigned from the GH10 library. However, we also identified three additional peaks from oligosaccharides of unknown structure which were named N_10_–N_12_. For the detailed structural characterisation of these oligosaccharides, the GH11 oligosaccharide mixture was loaded on a SEC column (Additional file 1: Figure S1 fractions *Ms*11_01 to *Ms*11_80) and unknown structures were consequently analysed by high-energy MALDI-CID.

The oligosaccharide N_10_ in the DASH trace was analysed from fraction *Ms*11_05. The [M + Na]^+^ ion at *m/z* 1012.8 corresponds to a Pent_5_ structure modified with one MeGlcA. The MALDI-CID reveals the structure of N_10_ as A^3^U^(4Me)2^XX (Additional file 1: Figure S8): The presence of the Y_1_ (*m/z* 294.3) and Y_2_ (*m/z* 426.3) ions indicate that the reducing end and the adjacent Xyl*p* are unsubstituted. The E_3_ ion (*m/z* 403.4) and H_3_ (*m/z* 435.3) sugar lactone indicate that the third Xyl*p* from the reducing end is modified at *O*-2 with a methylated glucuronic acid, while the V_3α_ product ion (*m/z* 936.3) indicates that the GlcA is modified with a methyl group at *O*-4. The series of Y_3_ (*m/z* 748.3) and Y_4_ (*m/z* 880.2) indicate that two pentosyl residues are present at the non-reducing end while the absence of the non-reducing end ^3,5^A_2_ ion (*m/z* 199.0) indicates that the non-reducing end Xyl*p* is substituted by a pentose. Finally the presence of the G_4_ reducing-end ion (*m/z* 846.3) is indicative of a substitution at *O*-3 of the reducing-end Xyl*p* residue. The N_10_ oligosaccharide was found sensitive to GH51 hydrolysis indicating that the pentosyl modification on the fourth Xyl*p* residue from the reducing end is an Ara*f* residue (Table 2).

The oligosaccharide N_11_ in the DASH trace was analysed from fraction *Ms*11_70. The [M + Na]^+^ ion at *m/z* 1144.8 corresponds to a Pent_6_ structure modified with one MeGlcA. The MALDI-CID combined with enzymatic hydrolysis was not conclusive but indicates two putative structures of N_11_ as B^2,3^U^(4Me)2^XX or D^2,3^U^(4Me)2^XX (Additional file 1: Figure S9).

The presence of the Y_1_ (*m/z* 294.3) and Y_2_ (*m/z* 426.3) ions indicated that the reducing end and the adjacent Xyl*p* are unsubstituted. The E_4_ ion (*m/z* 535.4) and H_4_ (*m/z* 567.3) sugar lactone indicate that the third Xyl*p* from the reducing end is modified at *O*-2 with a MeGlcA. This assignment is also confirmed by the presence of the G_3_ reducing-end ion (*m/z* 714.3). The V_3α_ product ion (*m/z* 1068.3) indicated that the GlcA was modified with a methyl group at the *O*-4 position. The presence of a series of Y ions, Y_4_ (*m/z* 880.2) and Y_5_ (*m/z* 1012.2), indicates that the reducing-end sugar sequence of this oligosaccharide is a linear pentose chain. However, the presence of G_4_ ion (*m/z* 846.3) indicated that the fourth Xyl*p* residue from the reducing end is modified at *O*-3. Furthermore the reducing-end ^0,2^X_4_ ion (*m/z* 1054.3) is indicative of a modification at *O*-2 of the fifth pentosyl group from the reducing end and therefore pointing to a structure similar to the D^2,3^XX (Fig. 5c). N_11_ oligosaccharide was resistant to *Cg*GH3 xylosidase which would be in accordance with the B^2,3^U^(4Me)2^XX assignment or could be explained by steric hindrance by MeGlcA modification on the adjacent Xyl*p* residue in the case of D^2,3^U^(4Me)2^XX assignment (Table 2).

The oligosaccharide N_12_ in the DASH trace was analysed from fraction *Ms*11_05. The [M + Na]^+^ ion at *m/z* 1276.9 corresponds to a Pent_7_ structure modified with one MeGlcA. The MALDI-CID combined with enzymatic hydrolysis reveals the structure of N_12_ as XA^3^XU^(4Me)2^XX (Additional file 1: Figure S10).

The series of Y ions (Y_1_, Y_2_, Y_3_, Y_4_ and Y_5_; *m/z* 294.4, *m/z* 426.4, *m/z* 748.3, *m/z* 880.3 and *m/z* 1144.2, respectively) indicate that the oligosaccharide is modified on the third Xyl*p* residue from the reducing end with [Me]GlcA and also on the fifth Xyl*p* residue from the reducing end with a pentosyl group. The presence of H_3_ (*m/z* 699.4) sugar lactone confirms that the third Xyl*p* residue is modified by a [Me]GlcA and indicates that this modification is at *O*-2. Additionally, the presence of G_5_ (*m/z* 978.3) reducing-end ions indicates that the fifth Xyl*p* residue is modified at *O*-3. The V_3α_ product ion (*m/z* 1200.4) indicates that the GlcA was modified with a methyl group at *O*-4. This oligosaccharide was found sensitive to GH51 and GH62 hydrolysis, indicating that the pentosyl modification on the fifth Xyl*p* residue from the reducing end is an Ara*f* residue, and to GH115 hydrolysis confirming that the uronic acid modification on the third Xyl*p* from the reducing end is a [Me]GlcA (Table 2 and Additional file 1: Figure S2A).

### Comparative structural analysis of GAX oligosaccharides derived from different grasses and different tissues

**Fig. 6:**
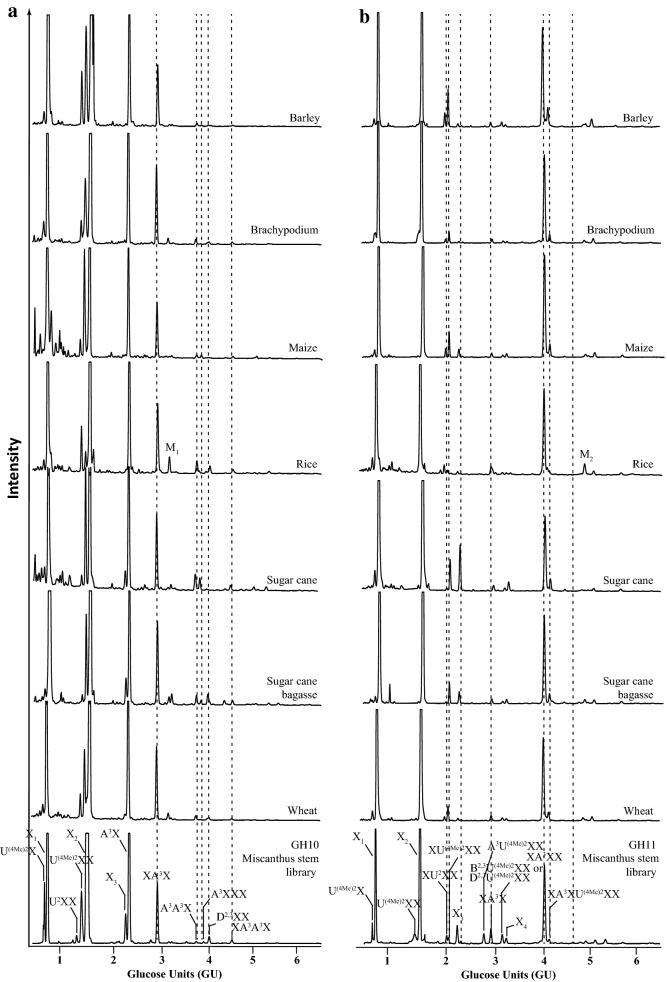
Comparison of DASH profiles of GAX oligosaccharides from stem of various grasses. **a** GH10 hydrolysis products; **b** GH11 hydrolysis products. Respective Miscanthus oligosaccharide library (bottom panel). Note the presence of two unknown peaks M_1_ and M_2_ in the rice DASH profile

**Fig. 7:**
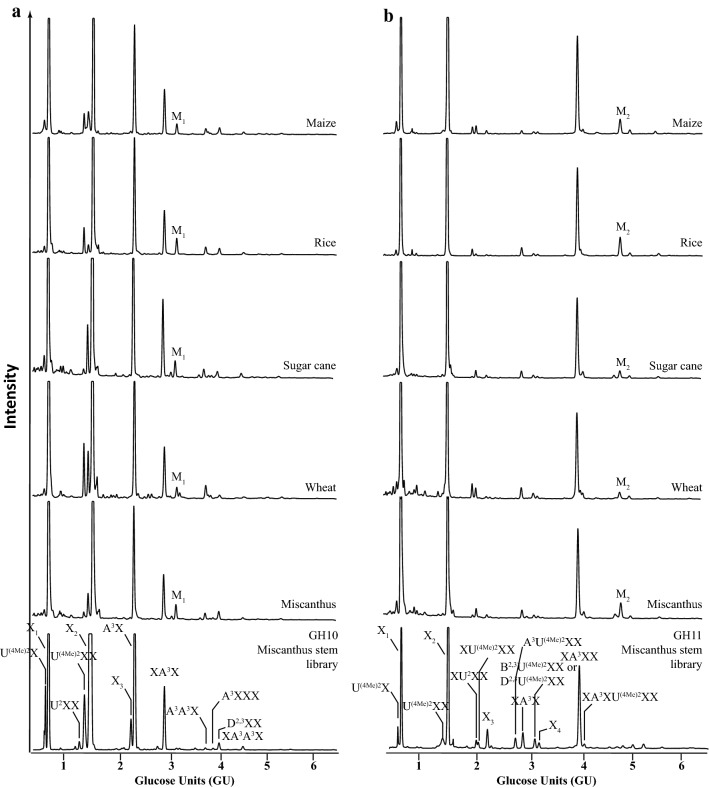
Comparison of DASH profiles of GAX oligosaccharides from leaves of various grasses. **a** GH10 hydrolysis products; **b** GH11 hydrolysis products. Respective Miscanthus oligosaccharide library (bottom panel). Note the abundance of the two unknown peaks M_1_ and M_2_ in profile of leaf material

The developed reference library was used to characterise variation of GAX in agriculturally important grasses in different aerial tissues. We analysed the xylooligosaccharide products of GH10 and GH11 hydrolysis from stems and leaves of brachypodium, maize, rice, sugar cane, wheat, leaves of miscanthus and stems of barley as well as sugar cane bagasse (Figs. 6 and 7).

The DASH profiles of the xylooligosaccharides of the GH10 and the GH11 digests of all grasses analysed look very similar and nearly all peaks can be annotated based on the GU of their oligosaccharides. The main peaks are X_1_, X_2_, A^3^X and XA^3^X, respectively. Overall, arabinosylation appears more abundant than glucuronidation and the latter seems more variable in abundance across grasses. In addition, the ratio of methylated and unmethylated GlcA seems to widely differ in grasses with rice GAX being rather unmethylated, whereas major proportion of GlcA in, e.g. miscanthus and sugar cane GAX is methylated. There is also a slight variance in abundance of the more complex oligosaccharides. Moreover, we can detect additional peaks: The most characteristic peaks, M_1_ and M_2_, are present in the DASH profiles of leaf GAX from all grasses analysed, but very rare in GAX of stem with the exception of rice stem GAX, in which M_1_ and M_2_ are substantial.

### Structural characterisation of the oligosaccharides M_1_ and M_2_ from leaf GAX

In order to identify the unknown oligosaccharides M_1_ and M_2_, maize leaf GH10 and rice leaf GH11 oligosaccharide products, respectively, were separated by SEC. The suitable SEC fractions (*Zm*10_65 for structure M_1_ and *Os*11_60 for structure M_2_) were further analysed by MALDI-ToF/ToF Mass Spectrometry, structural isomers were separated by HILIC and structurally characterised by high-energy MALDI-CID (Additional file 1: Figure S11).

The oligosaccharide M_1_ in the DASH trace was analysed from fraction *Zm*10_65 (Additional file 1: Figure S11A). The unknown oligosaccharide had an *m/z* 690.00 [M + Na]^+^, corresponding to a Pent_4_ structure (Additional file 1: Figure S11B). It did not co-migrate with Xyl_4_ in DASH, suggesting it is a substituted xylooligosaccharide. Off-line HILIC-MALDI-MS separated the two structural isomers with *m/z* 690.00 (M_1_ and XA^3^X, Additional file 1: Figure S11C) and the respective MALDI-CID spectra revealed the structure of M_1_ as D^2,3^X (Additional file 1: Figure S12): The presence of a series of Y ions (Y_1_, Y_2_ and Y_3_; *m/z* 294.3, 426.3 and 558.3, respectively) and the presence of ^1,4^X_2_ and ^1,5^X_3_ ions (*m/z* 454.3 and 586.3, respectively) indicate that the M_1_ is a linear DP4 pentose chain. The presence, however, of the G_3_ product ion (*m/z* 642.4, loss of 48 Da) indicates the existence of an Ara*f* residue in the structure. In addition, the presence of the G_4_ product ion (*m/z* 656.5) gives evidence of a terminal Xyl*p* residue on the structure. Finally, the presence of the ^0,2^X_2_ cross-ring ion (*m/z* 600.3) suggests that a pentose is 2-linked on the penultimate Xyl*p* residue. The assignment of M_1_ as D^2,3^X was confirmed by the finding that the structure is resistant to the hydrolysis with GH62 arabinosidase, GH67 and GH115 glucuronidases, but sensitive to *Cg*GH3 β-xylosidase hydrolysis (Table 2 and Additional file 1: Figure S2D).

The oligosaccharide M_2_ in the DASH trace was analysed from fraction *Os*11_60 (Additional file 1: Figure S11D). The unknown oligosaccharide had an *m/z* 954.5 [M + Na]^+^, corresponding to a Pent_6_ structure (Additional file 1: Figure S11E). It did not co-migrate with Xyl_6_ in DASH, suggesting it is a substituted xylooligosaccharide. Off-line HILIC-MALDI-CID revealed that this was the only structural isomer present in the sample (Additional file 1: Figure S11F). The MALDI-CID revealed the structure of M_2_ as XD^2,3^XX (Additional file 1: Figure S13): The significant Y_1_ ion (*m/z* 294.4) and the ^1,5^X_1_ ion (*m/z* 322.4) indicate that the 2-AA labelled Xyl*p* residue is not substituted. The Y_2_ and Y_3_ ions (*m/z* 426.4 and 690.4, respectively) show that the second Xyl*p* is unsubstituted but that the third Xyl*p* residue from the reducing end is modified with two pentose residues. The reducing-end G_3_ (*m/z* 524.5) product ion and the concomitant presence of the W_3_ (*m/z* 556.4) sugar lactone indicate that the substitution on the third Xyl*p* from the reducing end is at *O*-3. This assignment is further verified by the presence of the D_2_ (*m/z* 271.4) product ion. The existence of the G_4_ (*m/z* 906.5) product ion indicates the presence of an Ara*f* substitution. Finally, the presence of the ^3,5^A_2_ non-reducing end cross-ring fragment ion indicates the presence of an unsubstituted Xyl*p* at the non-reducing end. The assignment of M_2_ as XD^2,3^XX was confirmed by the finding that the structure is resistant to the hydrolysis with GH62 arabinosidase, GH67 and GH115 glucuronidases, but sensitive to *Cg*GH3 β-xylosidase hydrolysis (Table 2 and Additional file 1: Figure S2D).

### Relative quantitative differences in oligosaccharide structure and abundance in *Miscanthus sinensis* stem and leaf GAX

**Fig. 8:**
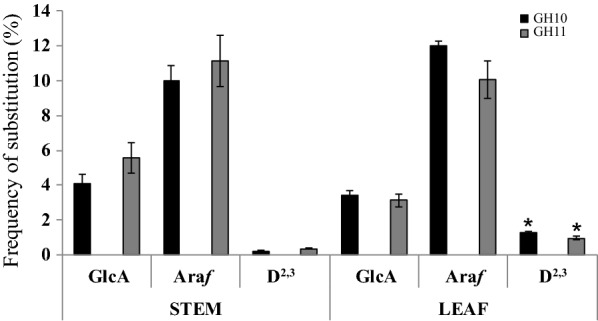
Quantification of substitution frequency of miscanthus stem and leaf GAX. All characterised GH10 and GH11 hydrolysis products were analysed by DASH using the DASHboard software for quantification. The substitution frequency was calculated after normalisation of values by comparing the abundance side chain to the Xyl*p* residues in the backbone. Error bars represent mean ± SD (*n* = 3). A significant difference was observed in the D^2,3^ substitution frequency of the GH10 digest (*p* = 0.002) and of the GH11 digest (*p* = 0.2) between stems and leaves and between the GH10 and G11 digest in leaves only (*p* = 0.01) using the paired t-test for two-tailed distribution, marked with asterisk

By DASH the relative quantity of reducing-end labelled oligosaccharides within a sample can be determined by the relative fluorescence intensity of the corresponding electropherogram peaks [27]. To analyse the difference in GAX structure in stems and leaves in greater detail, we quantified the frequency of substitution of all characterised library oligosaccharides in the GH10 and GH11 digests of three biological replicates of miscanthus using the DASHboard software (Fig. 8). The substitution frequency was calculated after normalisation of values by comparing the abundance of side chains to the Xyl*p* residues in the backbone.

First of all, the overall substitution frequency of xylan with [Me]GlcA, Ara*f* and D^2,3^ calculated from the two different digests with GH10 and GH11, respectively, is consistent with only a minor difference for the D^2,3^ structure in leaves, showing a slightly higher frequency in the GH10 digest only (1.3% versus 1%, *p* value 0.01).

In both, stems and leaves of miscanthus, arabinosylation of xylan is more frequent than glucuronidation, ranging between 10 and 12% of Ara*f* substitutions versus 3–6% of [Me]GlcA substitutions. However, there is a significant difference between tissues regarding the amount of D^2,3^ substitution both in the GH10 (*p* value 0.002) and the GH11 (*p* value 0.02) digests. In leaves the D^2,3^ substitution frequency of the GH10 and GH11 digest is 1.3% and 1%, respectively, whereas in stem it is down to 0.2% and 0.4%, respectively. Concomitantly, with the presence of the additional D^2,3^ structure (D^2,3^X and XD^2,3^XX) in leaf xylan, our data show that the frequency of the D^2,3^ structure is increased in leaves.

## Discussion

In this study, we used a combination of SEC, DASH, HILIC and MALDI-CID to elucidate the detailed structure of grass GAX oligosaccharides released by GH10 and GH11 xylanases. Based on these data, we developed a DASH reference library of structurally identified oligosaccharides and expressed their migration in GU. It has been reported previously that DASH can separate oligosaccharides with minor structural differences like methylation of GlcA [27]. Here, we show that DASH can successfully resolve structural isomers as demonstrated by the separation of the three DP5 oligosaccharides (*m/z* 822.0). Few approaches allow for wider screening of cell wall polysaccharide structures. Microarray-based glycan-profiling techniques [41, 42], for example, integrate the sequential extraction of glycans with the generation of microarrays, which are probed with monoclonal antibodies (mAbs) or carbohydrate-binding molecules (CBMs) with specificities for cell wall components. Although Microarray-based glycan profiling is a powerful technique offering high-throughput analysis of a wider variety of cell wall polymers, it is limited by the availability of mAbs and CBMs and their epitope specificity. A non-destructive high-throughput method for the compositional analysis of plant cell wall polymers is Fourier Transformed Mid-Infrared (FT-IR) spectroscopy [43]. This approach can provide structural information about substitution nature and frequency of cereal arabinoxylan [44]. The DASH reference library generated here allows the fast and robust comparison of a large number of GAX samples (96 samples in 50 min), providing detailed structural and quantitative information on biomass structural variation. Information gathered from DASH analysis will ultimately greatly facilitate the biorefining selection process of appropriate biomass.

We analysed the GAX structure of a number of different grass species (miscanthus, barley, brachypodium, maize, rice, sugar cane and wheat). The DASH profiles of all grasses analysed and the quantification data of xylan substitution of *Miscanthus sinensis* showed that Ara*f* side chains are more frequent than [Me]GlcA substitutions, which is consistent with sugar composition analysis of wheat straw [26, 45] and MS and NMR analysis of miscanthus, rice and brachypodium of the entire actively growing aerial portions [26]. Unlike cereal grain AX where 2-linked Ara*f* residues are abundant [1, 46–48], we did not detect any such substitutions, which is consistent with earlier reports on the structure of grass GAX in lignified tissues [24, 49, 50]. The overall xylan structure of the different grass species and of the different tissues is remarkably conserved, which is in line with earlier studies on grass xylan structure [51]. However, differences between species and tissues are detectable. Interestingly, the xylan structure of rice stems appears more similar to the xylan structure in leaves than to other xylan stem structures, forming an exception in the grasses analysed, perhaps because of the immature developmental stage of the culms collected. Plants exploit a number of variations in the xylan structure that were not studied in this work, e.g. the pattern of substitutions along the xylan backbone, feruloylation and coumaroylation of Ara*f* and acetylation of backbone Xyl*p*. These could be studied by exploitation of additional carbohydrate active enzymes and generation of additional standards in the DASH mobility library.

The most characteristic tissue-specific differences were identified in the form of the two oligosaccharides D^2,3^X and XD^2,3^XX, which are (apart from in rice) scarce in stem xylan but abundant in leaf xylan. Quantification of the overall frequency of the D^2,3^ side chain in miscanthus suggests that it is significantly more abundant in leaves than in stem. The D^2,3^ structure has been linked to feruloylation of xylan, and hence cross-linking and reduced digestibility [8]. Interestingly, the lignin amount and composition also differs in cell walls of miscanthus stems versus leaves [52]. Therefore xylan structural changes in different tissues might reflect specific levels and positioning of cross-linking, which can be a way of adjusting polymer structures to different functional requirements of the cell wall depending on its particular composition. A disaccharide side chain on xylan has been reported in sorghum [53] and switchgrass [54] although not detected by Bowman et al. [24] in a similar xylan analysis of switchgrass. The presence of this disaccharide side chain cannot be excluded although with our approach all disaccharide side-chain modifications positively identified were β-Xyl*p*-(1 → 2)-α-Ara*f*-(1 → 3) structures. The only exception was the GH11 product, putatively assigned as D^2,3^U^(4Me)2^XX, which was found resistant to *Cg*GH3 β-xylosidase. Resistance to *Cg*GH3 β-xylosidase could either be due to steric hindrance of the enzyme or could indicate the presence of an Ara*f*-(1 → 2)-α-Ara*f*-(1 → 3) side chain on this GH11 hydrolysis product (B^2,3^U^(4Me)2^XX). However, the Ara*f*-(1 → 2)-Ara*f*-(1 → 3)- adjacent to a Xyl*p* modified by a GlcA residue would be in a different substitution context to the Mazumder et al. [54] and Verbruggen et al. [48] reported oligosaccharides. If the latter is true then we could hypothesise that GH10 and GH11 xylanases hydrolyse slightly different parts of GAX. It is also, however, possible that the Ara*f*-(1 → 2)-α-Ara*f*-(1 → 3) side chain is not present in the tissues and plant species analysed here, or that it is present in amounts below the detection level.

It has been reported that the degree of methylation of GlcA on xylan varies between grass species and that in miscanthus methylation of xylan is more predominant than in, for example, rice or Brachypodium [49, 50]. This finding is consistent with our data on miscanthus xylan.

Some qualitative differences of oligosaccharides reflect enzyme specificity and distinct tolerance of the xylanases GH10 and GH11 to substitutions [28], for example, A^3^X from GH10 and XA^3^XX from GH11. However, some oligosaccharide such as A^3^U^(4Me)2^XX, D^2,3^U^(4Me)2^XX (or B^2,3^U^(4Me)2^XX) and XA^3^XU^(4Me)2^XX, although only minor products, were only detectable in GH11 digestion and might indicate that GH10 and GH11 act on different domains of grass xylan as they resemble different substitution patterns. The fact that the level of 3-linked Ara*f* and [Me]GlcA substitutions is remarkably similar independent of the xylanase used does not necessarily support this hypothesis. Surprisingly, some of these minor digestion products of GH11 harbour substitutions at the non-reducing end of the oligosaccharide, which are not predicted as products and might be the result of either using the enzymes in excess or that the GH11 enzyme preparation used in this study was contaminated with small amounts of either GH10 xylanase or β-xylosidase. Alternatively, these oligosaccharides could derive from the non-reducing end of the xylan polymer and would be produced by a single cleavage at the reducing end of the oligosaccharide.

## Conclusions

As characterised here in grass stems and leaves from several species, mainly three GAX sugar side chains, 3-linked Ara*f*, 2-linked GlcA/MeGlcA and 3-linked Xyl*p*-(1 → 2)-Ara*f*, are utilised to decorate the xylan molecule. Our GAX oligosaccharide reference library of DASH mobilities, developed in this study, provides a means to study structural aspects of xylan and might help to shed light on how structural changes of xylan correlate with the interaction of this polysaccharide with other cell wall components, how this influences its biological function, its mechanical properties and recalcitrance of the cell wall. Furthermore it provides a high-throughput quantitative method for the selection of suitable lignocellulosic biomass and tailoring of biorefining processes.

## Methods

### Plant material

The plant materials used in this study were fresh material from *Miscanthus sinensis* (miscanthus; Wageningen University, Netherlands, Luisa Trindade & Oene Dolstra), *Hordeum vulgare* (barley; University of Dundee, Claire Halpin), *Brachypodium distachyon* (Brachypodium; (grown at University of Cambridge greenhouse), *Zea mays* (maize; University of Cambridge, Paul Dupree), *Oryza sativa* (rice; grown at University of Cambridge greenhouse), *Saccharum officinarum* (sugar cane; EMBRAPA-Brazil, Christiane Farinas), sugar cane bagasse (University of Sao Paulo-Brazil, Marcos Buckeridge) and *Triticum aestivum* (wheat; University of Nottingham, Greg Tucker).

### Extraction of alcohol-insoluble residue (AIR)

Plant stems and leaves were harvested, submerged in 96% (v/v) ethanol and boiled at 70 °C for 30 min to inactivate enzymes. Following homogenisation using a ball mixer mill (Glen Creston), the pellet was collected by centrifugation (4000 x g for 15 min) and was washed with 100% (v/v) ethanol, twice with chloroform:methanol (2:1), followed by successive washes with 65% (v/v), 80% (v/v) and 100% (v/v) ethanol. The remaining pellet of AIR was air dried. Aqueous suspensions (0.5 mg/ml; at 21 °C) of AIR were prepared using a glass homogeniser and kept for further analysis.

### Hemicellulose extraction

Hemicelluloses were extracted by treating AIR preparations (2 g for Size exclusion chromatography (SEC) fractionation; 50 μg for small-scale digestions) with a small volume of 4 M NaOH (5 ml for SEC fractions; 20 μl for small-scale digestions) for 1 h at room temperature before the pH was adjusted to about pH 6.0 with 1 N HCl; 50 mM ammonium acetate buffer pH 6.0 was added (200 ml for SEC fractionation; 0.5 ml for small-scale digestions). Note: Alkali treatments results in the removal of acetylation and feruloylation.

### Enzymatic hydrolysis and enzymes

Glycoside hydrolases of different Carbohydrate Active enZYme families were used in this study: GH10 endo-β-1,4-xylanase *Cj*GH10A from *Cellvibrio japonicus* [55]; GH11 endo-β-1,4-xylanase *Np*GH11A from *Neocallimastix patriciarum* [56]; GH67 α-glucuronidase *Cj*GH67 from *Cellvibrio japonicus* [37, 57] and GH115 α-glucuronidase *Bo*GH115 from *Bacteroides ovatus* [38, 58]; GH62 α-arabinofuranosidase *Pa*GH62 from *Penicillium aurantiogriseum* [59], a gift from Novozymes; GH51 α-arabinofuranosidase *Pc*GH51 from *Pseudomonas cellulose* [36]; GH3 xylosidase *Cg*GH3 from *Chaetomium globosum* (NS39127) and GH3 β-1,4 xylosidase *Tr*GH3 from *Trichoderma reesei* [60] were both gifts from Novozymes. All enzymes were added at a final concentration of 2 μM and incubated at 21 °C under constant shaking for 24 h. Enzymatic hydrolysis progression was monitored by Polysaccharide Analysis Using Carbohydrate Gel Electrophoresis [61] and if necessary, the enzyme amount was adjusted to ensure complete hydrolysis.

### Enzyme deactivation and removal of undigested material

Enzymes were then deactivated by boiling for 30 min undigested cell wall material was removed by centrifugation. In case of SEC, centrifugation was followed by filtration (Whatman 45 μm). Samples were then dried in a centrifugal evaporator.

### Size exclusion chromatography

Dried hydrolysed cell wall materials (corresponding to 2 g of AIR) were resuspended in 2 ml distilled water. Sample (2 ml) was applied onto the column and eluted with distilled water. SEC was performed on a gravity-driven Bio-Gel P-2 column (190 × 2.5 cm, BioRad) as previously described [62]. 2 ml fractions were collected, concentrated to 100 μl in a centrifugal evaporator and 10 μl were analysed by DASH as described below. A total of 80 SEC fractions were collected for each grass species and xylanase hydrolysis analysed. The SEC fraction naming system includes information on the species (*Ms: Miscanthus sinensis*, *Zm: Zea mays* and *Os: Oryza sativa*), the xylanase (10: GH10 and 11: GH11) and the fraction number (_01 to _80). The fractions with xylooligosaccharides of interest were subjected to secondary enzymatic hydrolysis or dried, desalted, reductively aminated with 2-anthranilic acid (2-AA), purified by HILIC and structurally characterised by high-energy MALDI-CID.

### APTS labelling and analysis by DASH

The derivatisation of oligosaccharides with 8-aminopyrene-1,4,6-trisulfonate (APTS) was performed according to previously developed protocols [27]. A set of 7 fluorophore (DY-481XL-NHS ester)-labelled amino acids and peptides was used as electrophoretic mobility standards (Asp–Asp–Asp–Asp; Asp–Asp–Asp; Glu–Glu; Cysteic acid; l-2-Aminoadipic acid; Glycine; Gly–Gly–Gly) to align the electropherograms. These electrophoretic mobility standards were mixed with each sample prior to DASH separation serving as internal mobility markers. DASH data generated by the DNA sequencer were processed with the DASHboard software [27]. Control experiments without the substrates were performed under the same conditions in order to identify any non-specific compounds in the enzymes or labelling reagents. An APTS-derivatised dextran ladder (0.1 M TFA hydrolysis at 100 °C for 2 h; 50 μg μl^−1^ dextran in 200 μl TFA solution) was simultaneously separated by DASH and was used to provide the GU migration positions.

### Desalting and clean up for HILIC separation

Xylooligosaccharides from SEC fractions were desalted using HyperSep Hypercarb cartridges (Thermo-Hypersil-Keystone, Runcorn, Cheshire, UK) as previously described [29]. Oligosaccharides were lyophilised and then derivatised with 2-aminobenzoic acid (2-AA) as described below.

### Reductive amination and purification for HILIC separation

SEC-purified xylooligosaccharides were reductively aminated with 2-AA (Sigma) and then purified from the reductive amination reagents using a Glyko Clean S cartridge (Prozyme, San Leandro, CA) as previously described [63].

### HILIC-MALDI-MS and MALDI-MS/MS CID analysis

Capillary HILIC was carried out using an LC-Packings Ultimate system (Dionex, CA, USA) equipped with an amide-80 column (300 μm × 25 cm; 3 μm particle size; Dionex) as previously described [63]. Briefly, the LC system was used to generate the gradient that flowed at 3 μl min^−1^. Solvent A was 50 mm ammonium formate adjusted to pH 4.4 with formic acid. Solvent B was 5% solvent A in acetonitrile. The labelled oligosaccharides dissolved in 95% acetonitrile were loaded onto the column (20 μl) and eluted with increasing aqueous concentrations. The following gradient conditions were applied: 0 min, 5% solvent A, 95% solvent B; 6 min, 25% solvent A, 75% solvent B; 86 min, 45% solvent A, 55% solvent B. The system operated at ambient temperature. The column eluent passed through a capillary UV detector (set at 254 nm) to the MALDI sample spotter. For HILIC-MALDI-ToF/ToF Mass Spectrometry, a Probot sample fraction system (Dionex) was employed for automated spotting of the HPLC eluent onto a MALDI target at 20 s intervals. After air drying, the sample spots were overlaid with 0.5 μl 2,5-DHB matrix (1 mg ml^−1^ in 50% aqueous methanol) and analysed by MALDI-ToF/ToF–MS on an AB-Sciex 4700. The MS spectra were obtained in automatic mode with an average 1500 laser shots/spectrum (mass range 400–2500 Da). The oligosaccharide molecular ions [M + Na]^+^ were identified in the MALDI data and their HILIC elution positions were determined by carrying out an extracted ion chromatogram (EIC). High-energy MALDI-CID spectra were acquired with an average 10,000 laser shots/spectrum, using a high collision energy (1 kV). The oligosaccharide ions were allowed to collide in the CID cell with argon at a pressure of 2 × 10^−6^ Torr.

### NMR analysis

Saponified miscanthus AIR was hydrolysed with GH10 xylanase, followed by GH115 xylan glucuronidase, GH51 arabinofuranosidase and *Tr*GH3 β-1,4-xylanase using enzyme hydrolysis conditions described above. The resulting N_8_ oligosaccharide was then isolated by SEC as described above. Consequently, SEC fractions containing the N_8_ oligosaccharide were pooled, solubilised in 0.6 ml D_2_O and analysed by NMR.

NMR spectra were recorded at 298 K with a Bruker AVANCE III spectrometer operating at 600 MHz equipped with a TCI CryoProbe. Two-dimensional ^1^H-^1^H TOCSY, ROESY, ^13^C HSQC and HSQC-TOCSY experiments were performed, using established methods [64]; the mixing times were 70 ms and 200 ms for the TOCSY and ROESY experiments, respectively. Chemical shifts were measured relative to internal acetone (δH = 2.225, δC = 31.07 ppm). Data were processed using the Azara suite of programs (v. 2.8, copyright 1993–2014, Wayne Boucher and Department of Biochemistry, University of Cambridge, unpublished) and chemical-shift assignment was performed using Analysis v2.2 [65].

### Oligosaccharide naming system

The various hydrolysis products are named according to the Faure et al. [30] naming system. This naming system utilises a single letter code where the uppercase letters identify the substituents of the main xylan chain. The letter “A” is attributed to single Ara*f* substitution, the letter “U” is used for single GlcA substitution and the letter “X” for unsubstituted Xyl*p* residues. The superscript numbers indicate substitution linkage position on Xyl*p*. Information of side-chain modifications is included in the superscript part of the name, for example, “Me” for methylation. Finally, further substitutions of the side chains receive a new letter assignment, for example, the Ara*f*-(1 → 2)-α-Ara*f*-(1 → 3) side chain has been designated the “B^2,3^” character and the β-Xyl*p*-(1 → 2)-α-Ara*f*-(1 → 3) substitution has been designated the “D^2,3^” character [30].

### DASHboard software and substitution frequency quantitation

Data generated by the DNA sequencer were processed in DASHboard software [27] which was developed to complement the profiling technique and perform tasks such as visualisation of data, alignment of electropherograms, peak area quantification and export to Excel for further analysis.

Relative quantitation of substitution frequency was calculated after normalisation of values by comparing the abundance of side chains to the Xyl*p* residues in the backbone. Value normalisation allowed the accurate peak area calculation by DASH software because electropherograms from higher dilution were utilised for the peak area calculation of highly abundant mono- and oligosaccharides (such as xylose and xylobiose) and the electropherograms of same samples but of lower dilution were utilised for peak area calculation of less abundant oligosaccharides. The peak area ratio between each of the xylanase oligosaccharide products and a reference oligosaccharide (XA^3^X and XA^3^XX for GH10 and GH11 digestions, respectively) resulted in the normalised values for each oligosaccharide. The total backbone Xyl*p* present in the digested xylan was calculated as the sum of the relative quantity of each of these digestion products multiplied by the number of Xyl*p* residues present in each structure (total backbone Xyl*p*). For each specific side chain, side-chain substitution was calculated as the sum of the relative quantity of each of these side chains (Ara*f*, [Me]GlcA and D^2,3^) multiplied by the number of side chains present in each structure (side-chain substitution). Hence, side-chain substitution frequency was calculated as the ratio between (side-chain substitution) and (total backbone Xyl*p*).

## Additional files

**Additional file 1.** Additional figures.

**Additional file 2: Table S1.** ^1^H and ^13^C NMR assignments of β-Xyl*p*-(1 → 2)-α-Ara*f*-(1 → 3)-β-Xyl*p*-(1 → 4)-β-Xyl*p*-(1 → 4)-β-Xyl*p*, at 25 °C in D_2_O.

## Abbreviations

DASH: DNA sequencer-Assisted Saccharide analysis in High throughput
Ara*f*: arabinofuranose/arabinofuranosyl
Xyl*p*: xylopyranose/xylopyranosyl
[Me]GlcA: [methyl]glucuronic acid/glucuronyl
(G)AX: (glucurono)arabinoxylan
AIR: alcohol-insoluble residue
GH: glycosyl hydrolase
GU: glucose units
