# Supplementary material for: Development of an oligosaccharide library to characterise the structural variation in glucuronoarabinoxylan in the cell walls of vegetative tissues in grasses
Source: Biotechnol Biofuels. 2019 May 6;12:109. doi: 10.1186/s13068-019-1451-6 (PMC6501314; doi:10.1186/s13068-019-1451-6)
Supplement: Supplementary file 1 — Additional file 1. Additional figures. [file 13068_2019_1451_MOESM1_ESM.docx]

*Additional Figures and Figure captions*

**
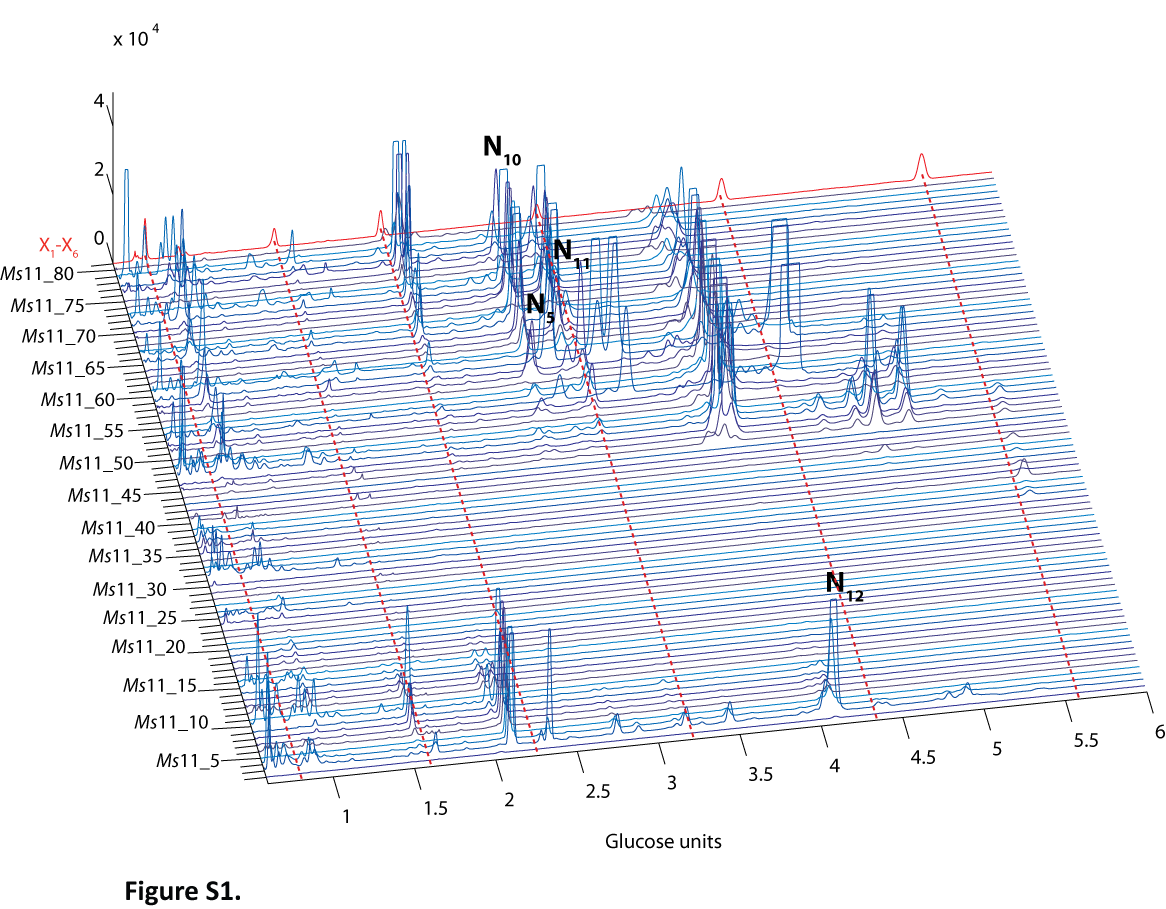
**

**Figure S1.** DASH profile of SEC fractions from hydrolysis of miscanthus stem. 80 SEC fractions were separated and analysed by DASH (blue and grey traces), xylooligosaccharide standards X_1_-X_6_ (red trace).

**
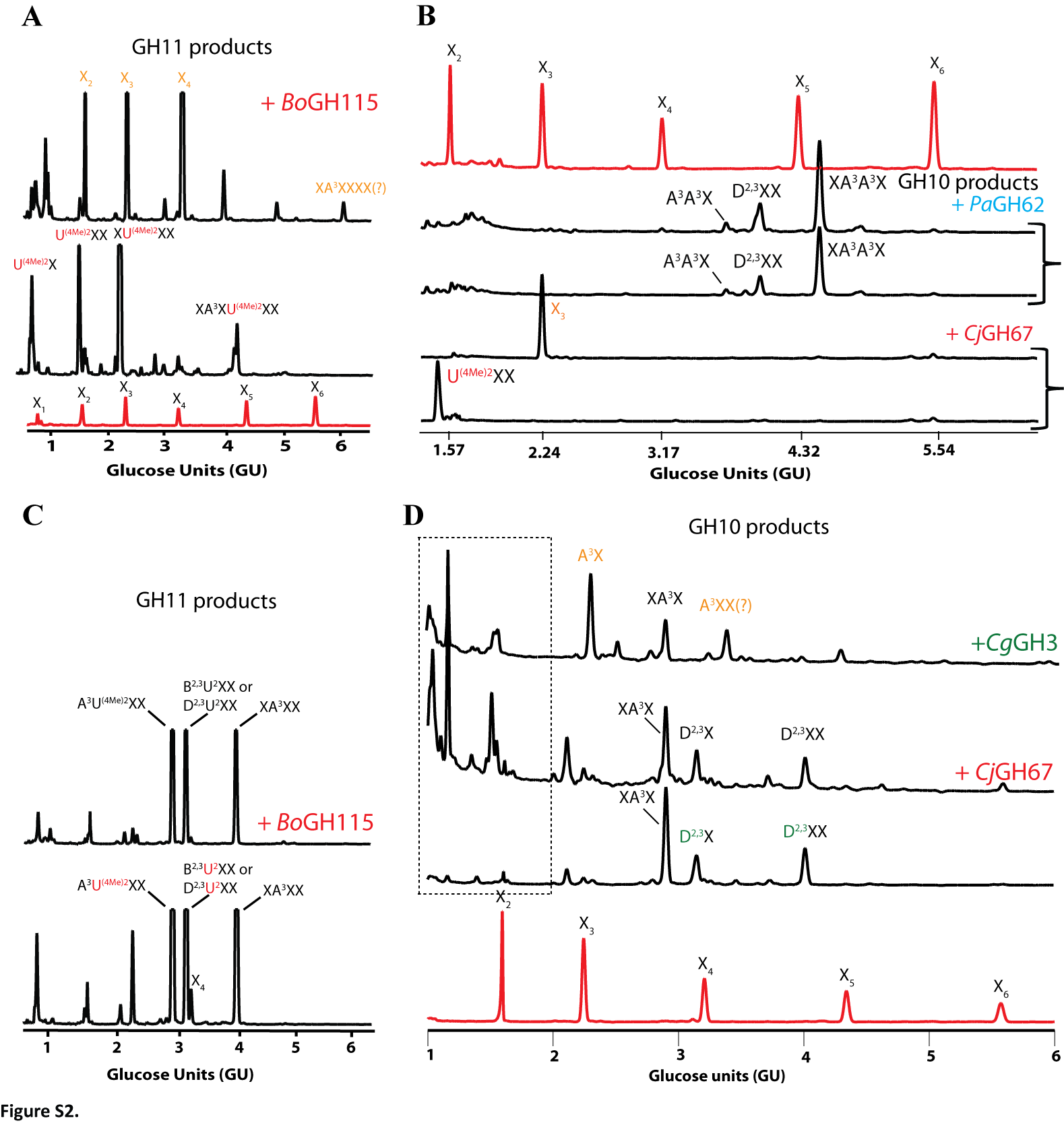
**

**Figure S2.** Structural characterisation of Miscanthus stem xylooligosaccharides by enzyme hydrolysis. Xylooligosaccharide products were analysed by DASH. The oligosaccharides sensitive to glucuronidases *Bo*GH115 or *Cj*GH67 are highlighted in red, oligosaccharides sensitive to *Cg*GH3 are highlighted green and oligosaccharides sensitive to arabinofuranosidase *Pa*GH62 are highlighted in blue. Resulting products are highlighted in orange, oligosaccharides that are not sensitive to an enzymatic treatment are presented in black ink. Xylooligosaccharides, X_1_-X_6_ are used as standard. Area marked with grey dashed box corresponds to background noise. Note that because of the loss in charge after removal of the GlcA side chain oligosaccharides elute later by DASH as the mobility is reduced. (A) Comparison of DASH profiles of GH11 products from SEC fraction *Ms*11_05 and with and without *Bo*GH115 for the characterisation of U^(4Me)2^X, U^(4Me)2^XX, XU^(4Me)2^XX, A^3^U^(4Me)2^XX and XA^3^XU^(4Me)2^XX oligosaccharides. (B) Comparison of DASH profiles of GH11 products from SEC fraction *Ms*11_70 and sequential hydrolysis of *Ms*11_70-oligosaccharides with *Bo*GH115 for the characterisation of A^3^U^(4Me)2^X, B^2,3^U^2^XX or D^2,3^U^2^XX, XA^3^XX oligosaccharides. (C) Comparison of DASH profiles of GH10 products from SEC fraction *Ms*10_60 and sequential hydrolysis of *Ms*10_60-oligosaccharides with *Pa*GH62 for the characterisation of A^3^A^3^X, D^2,3^XX and XA^3^A^3^X; from SEC fraction *Ms*10_40 and sequential hydrolysis of *Ms*10_40-oligosaccharides with *Cj*GH67 for the characterisation of U^(4Me)2^XX. (D) Comparison of DASH profiles of GH10 products from SEC fraction *Zm*10_65 and sequential hydrolysis of *Zm*10_65-oligosaccharides with *Cj*GH67 or *Cg*GH3 for the characterisation of D^2,3^X and D^2,3^XX.

**
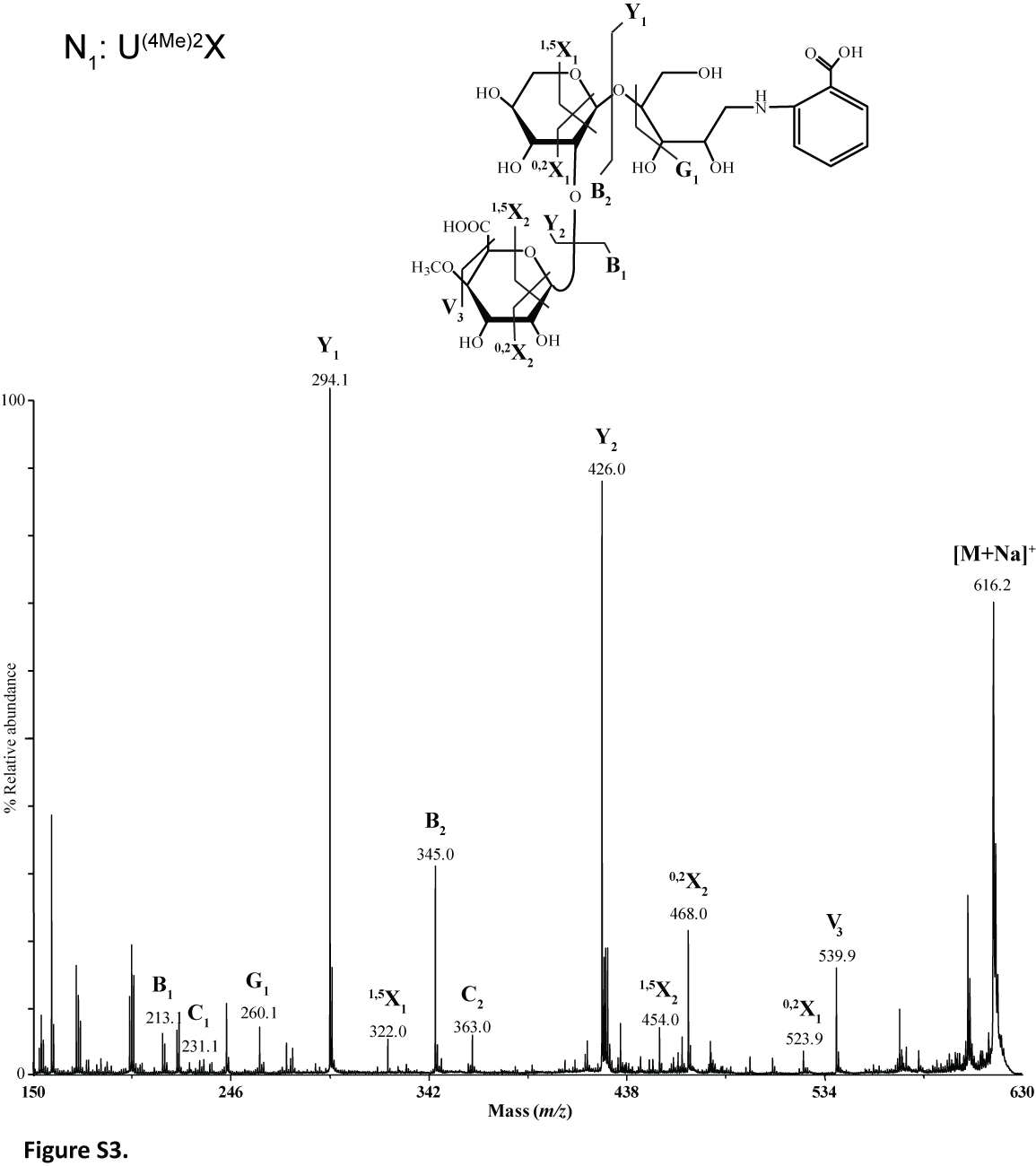
**

**Figure S3.** High energy MALDI-CID spectrum of the N_1_ structure: U^(4Me)2^X.


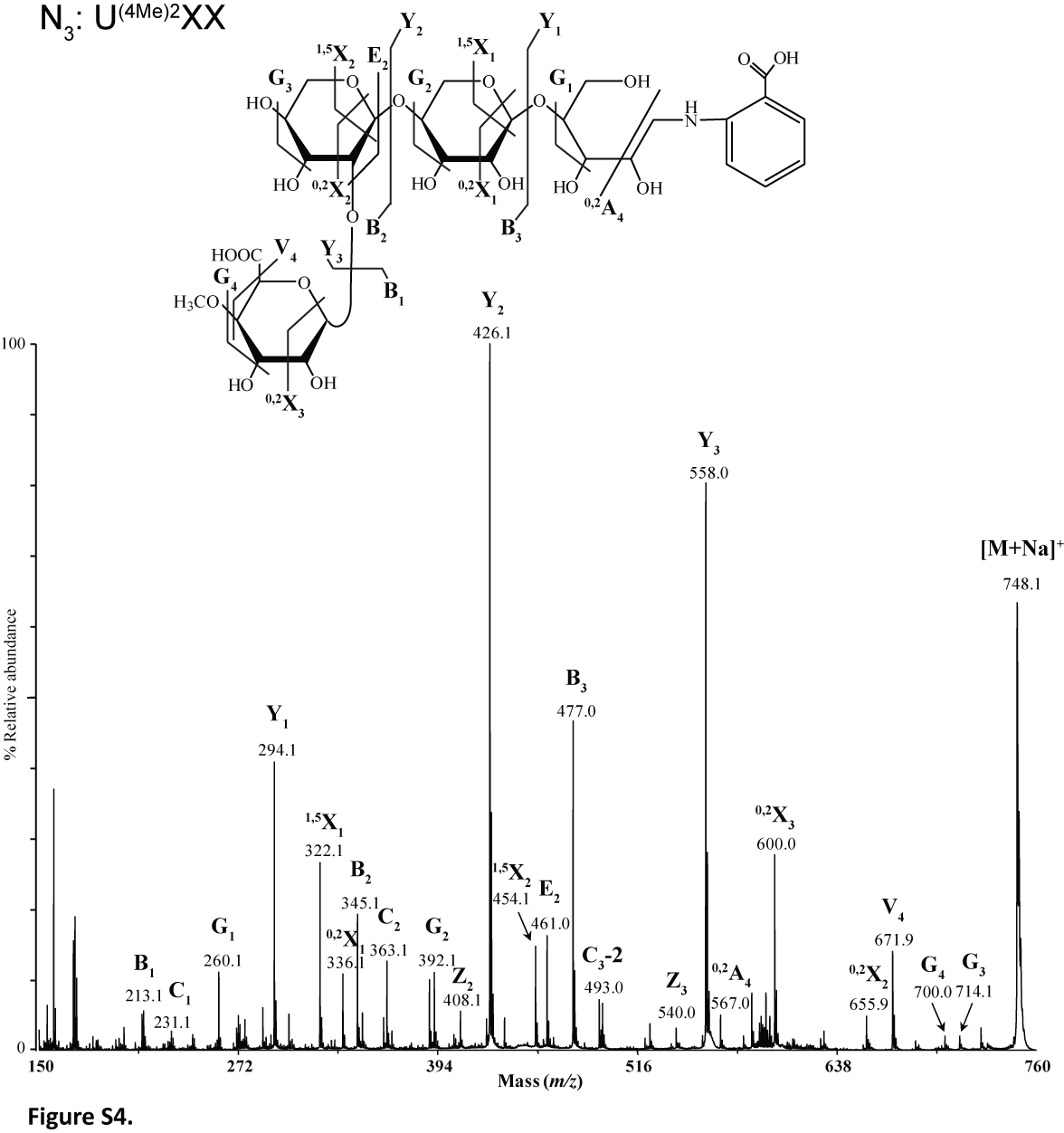


**Figure S4.** High energy MALDI-CID spectrum of the N_3_ structure: U^(4Me)2^XX.


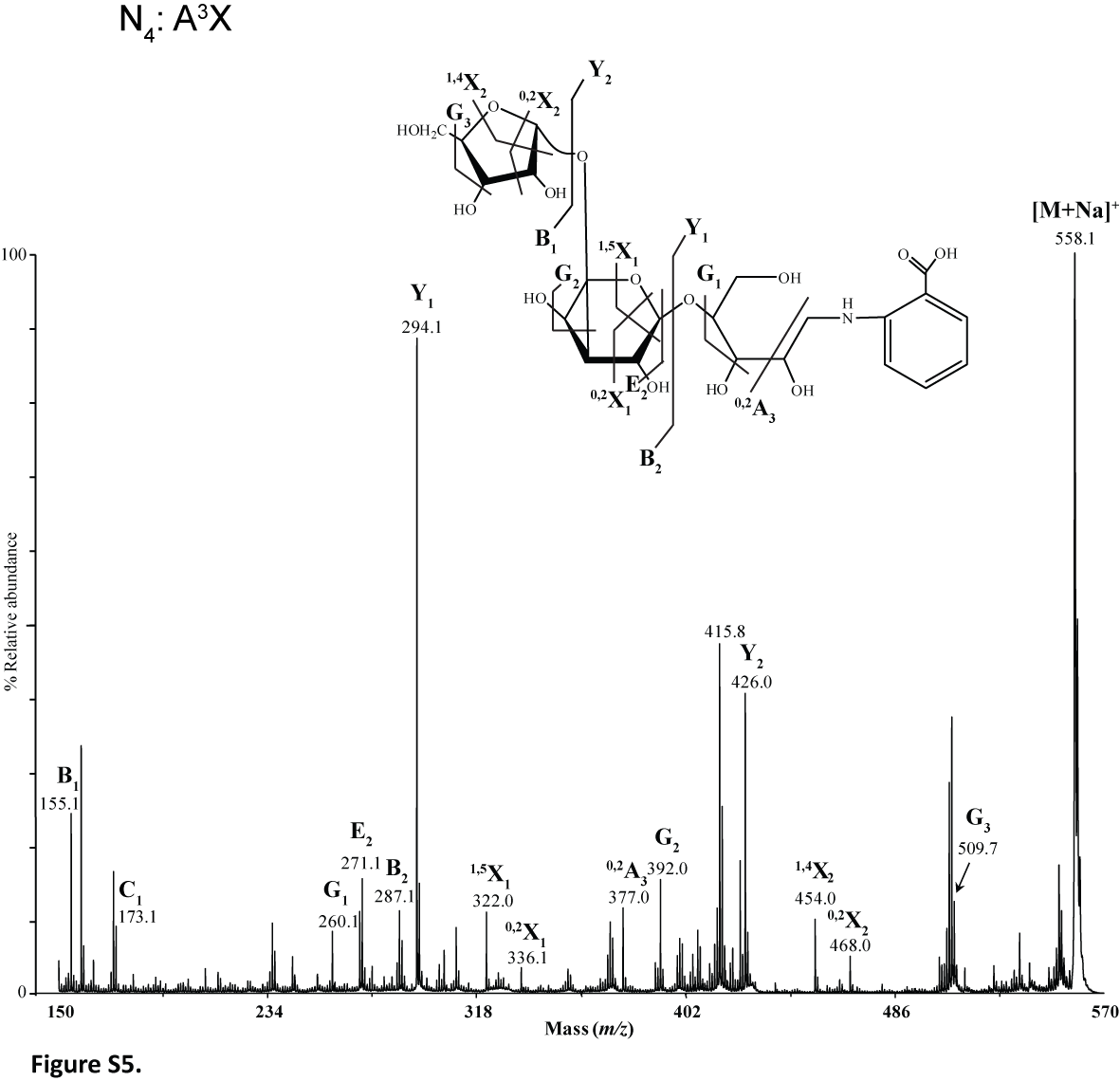


**Figure S5.** High energy MALDI-CID spectrum of the N_4_ structure: A^3^X.


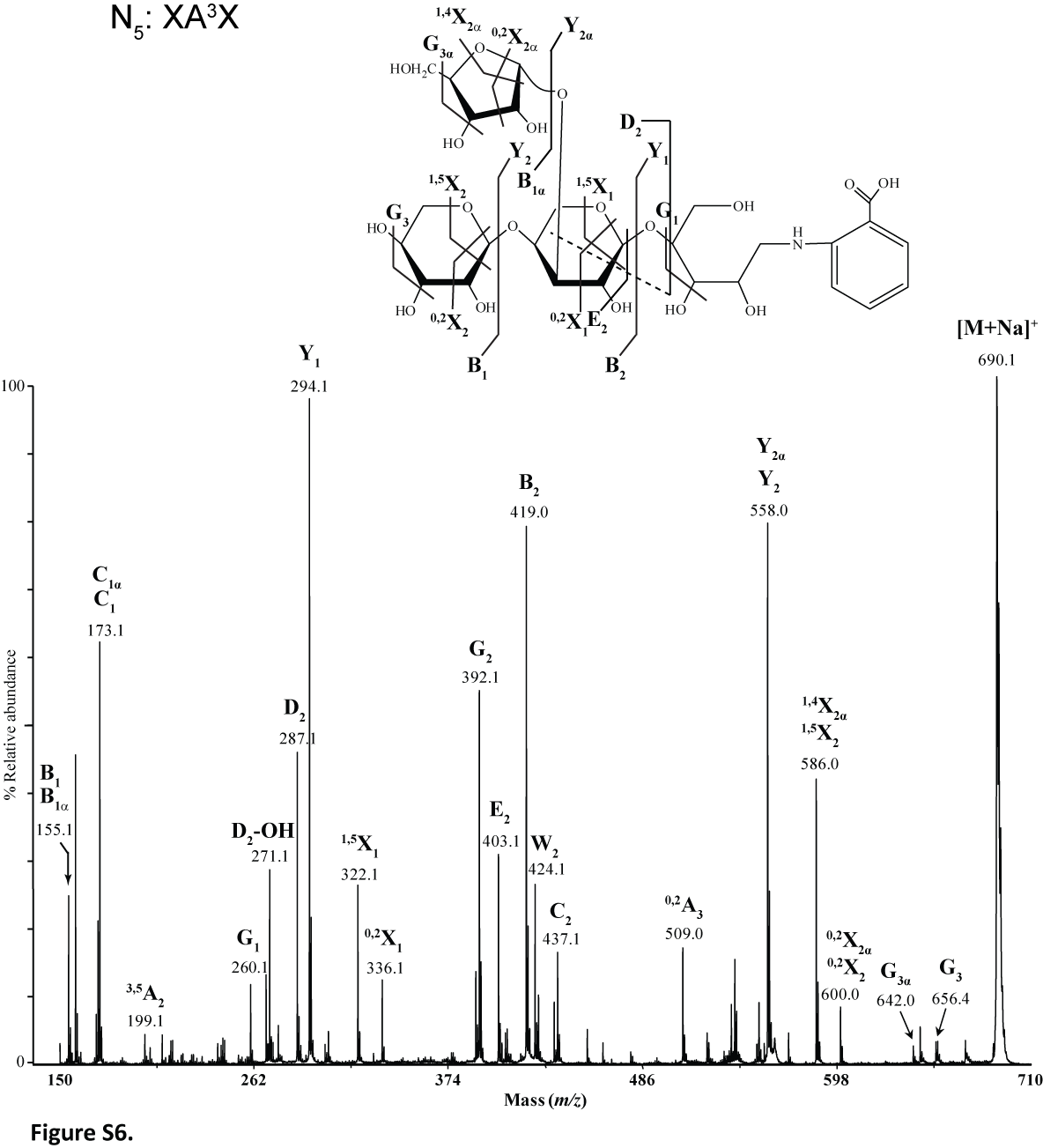


**Figure S6.** High energy MALDI-CID spectrum of the N_5_ structure: XA^3^X. Note: The proposed chemical structure for W product ion can be seen in Figure 4A.

**
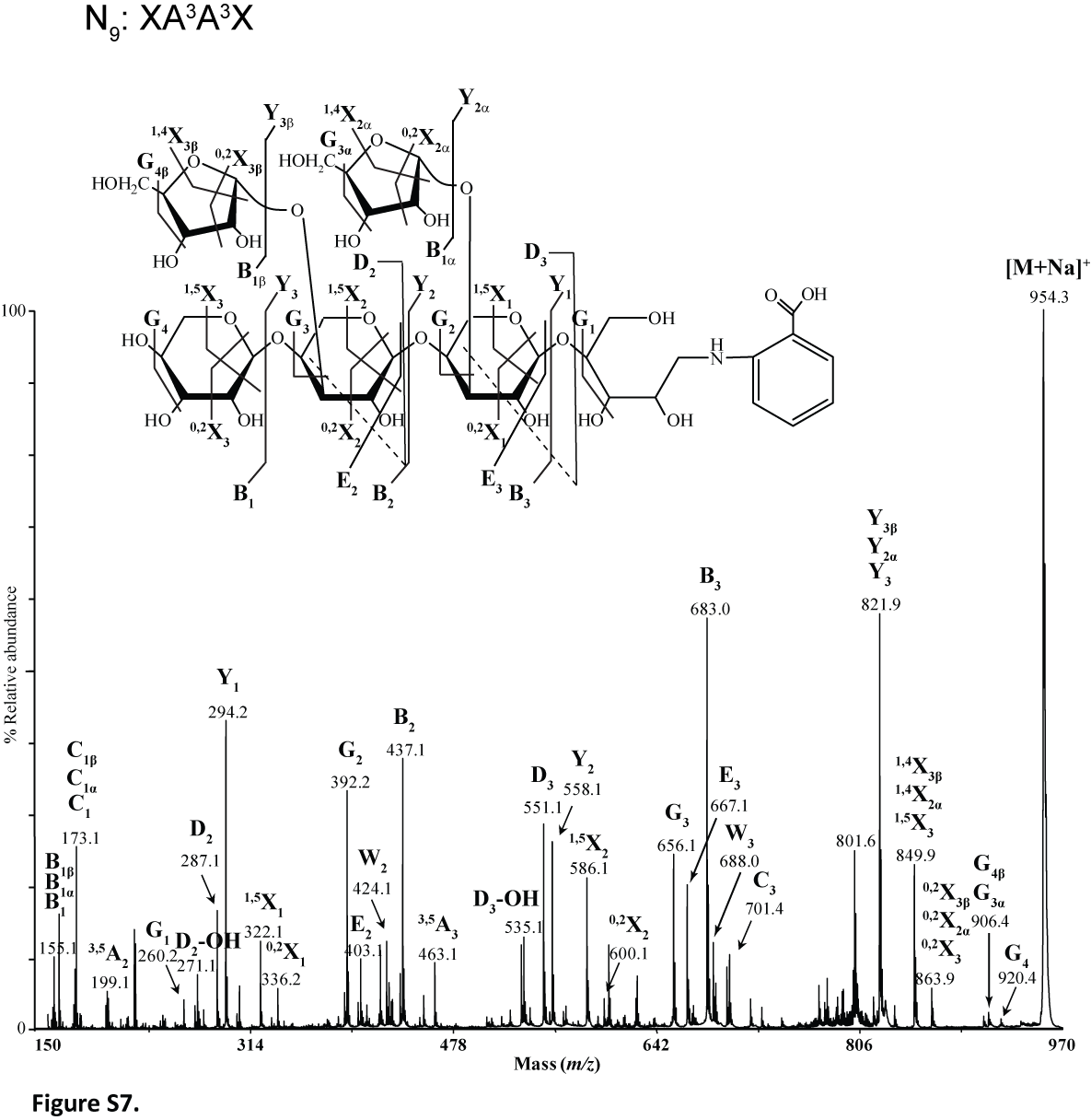
**

**Figure S7.** High energy MALDI-CID spectrum of the N_9_ structure: XA^3^A^3^X.


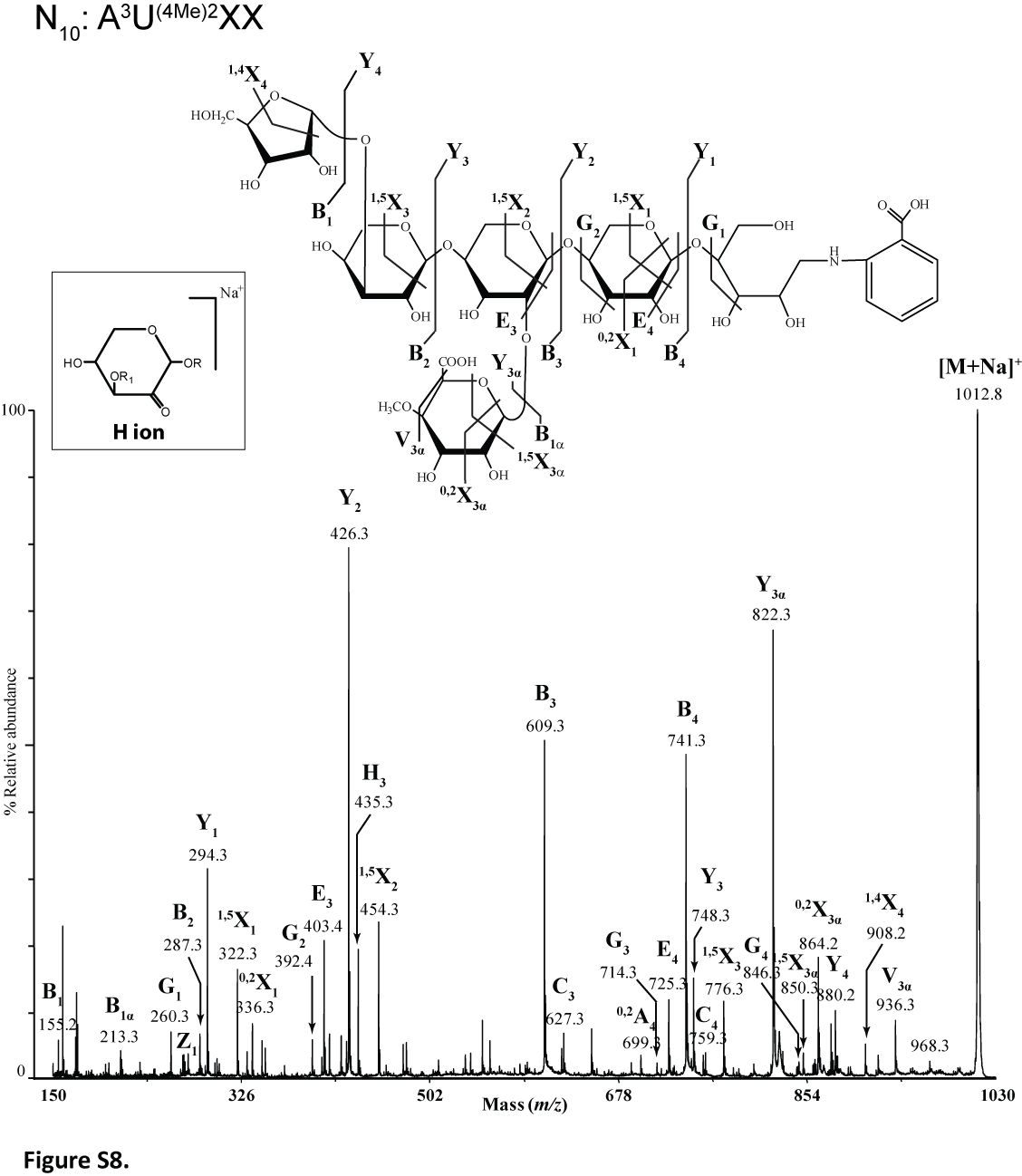


**Figure S8.** High energy MALDI-CID spectrum of the N_10_ structure: A^3^U^(4Me)2^XX. Inset: proposed chemical structure for H product ion (Maslen et al., 2007)


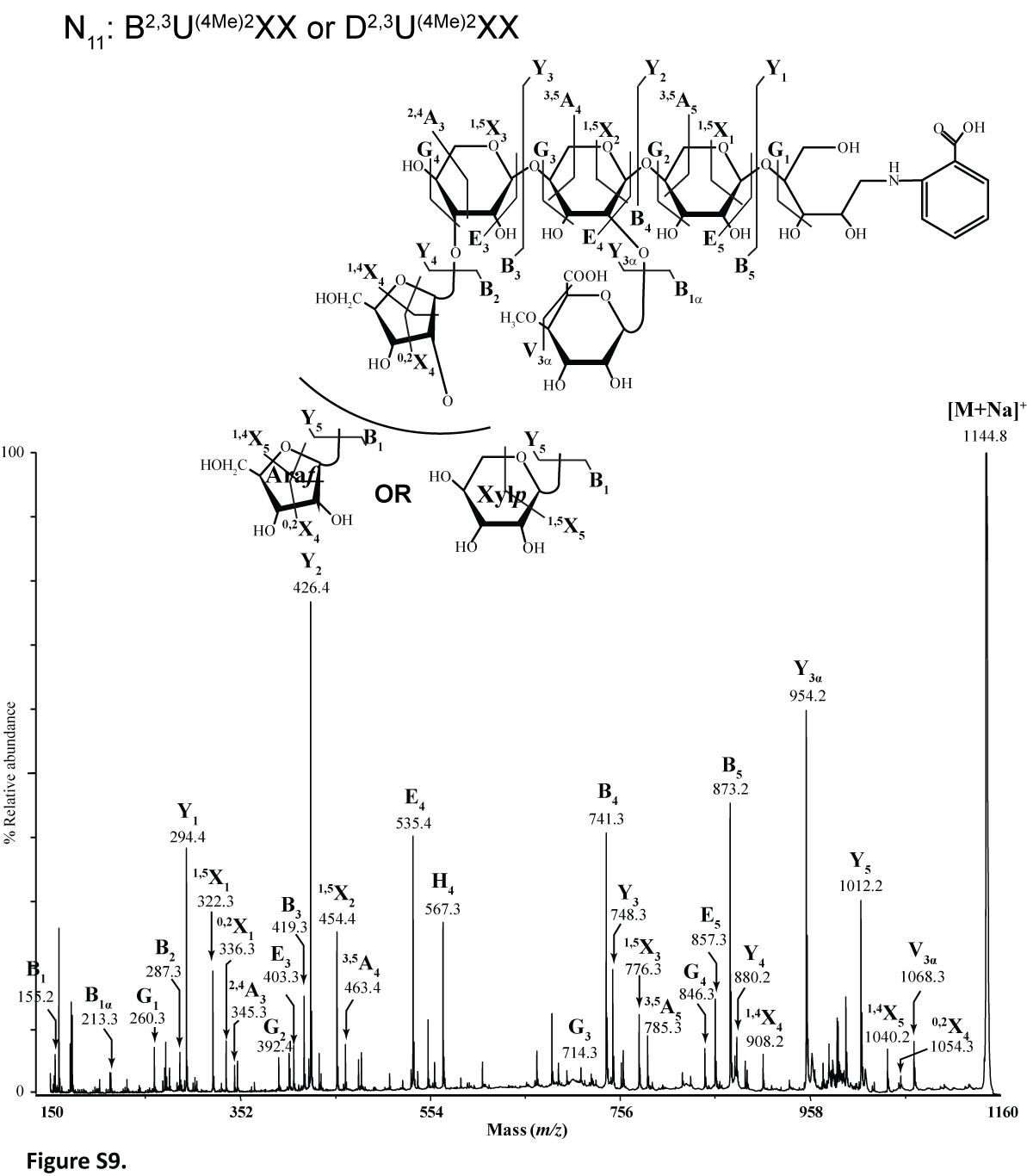


**Figure S9.** High energy MALDI-CID spectrum of the N_11_ structure: B^2,3^U^(4Me)2^XX or D^2,3^U^(4Me)2^XX.


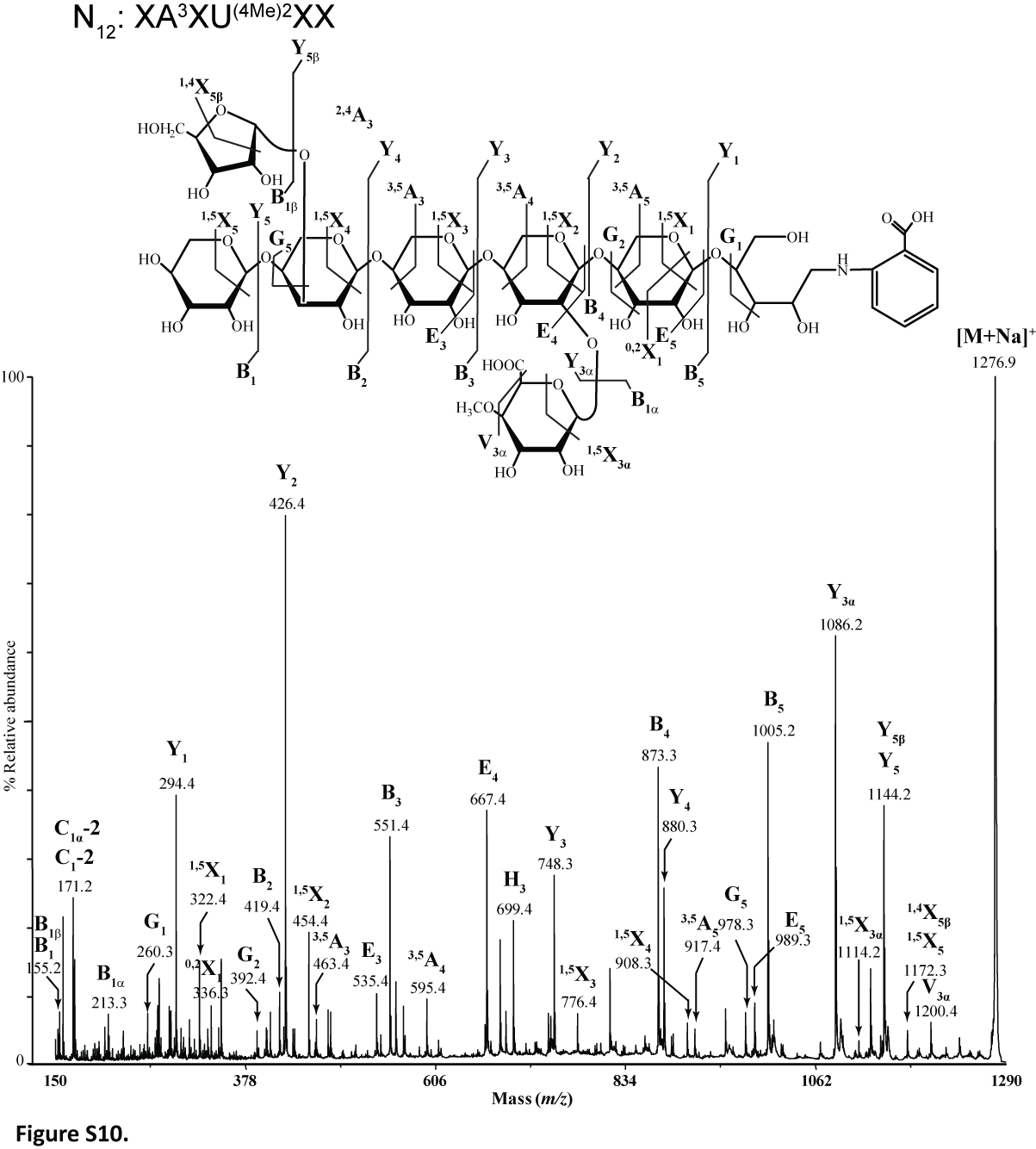


**Figure S10.** High energy MALDI-CID spectrum of the N_12_ structure: XA^3^XU^(4Me)2^XX.


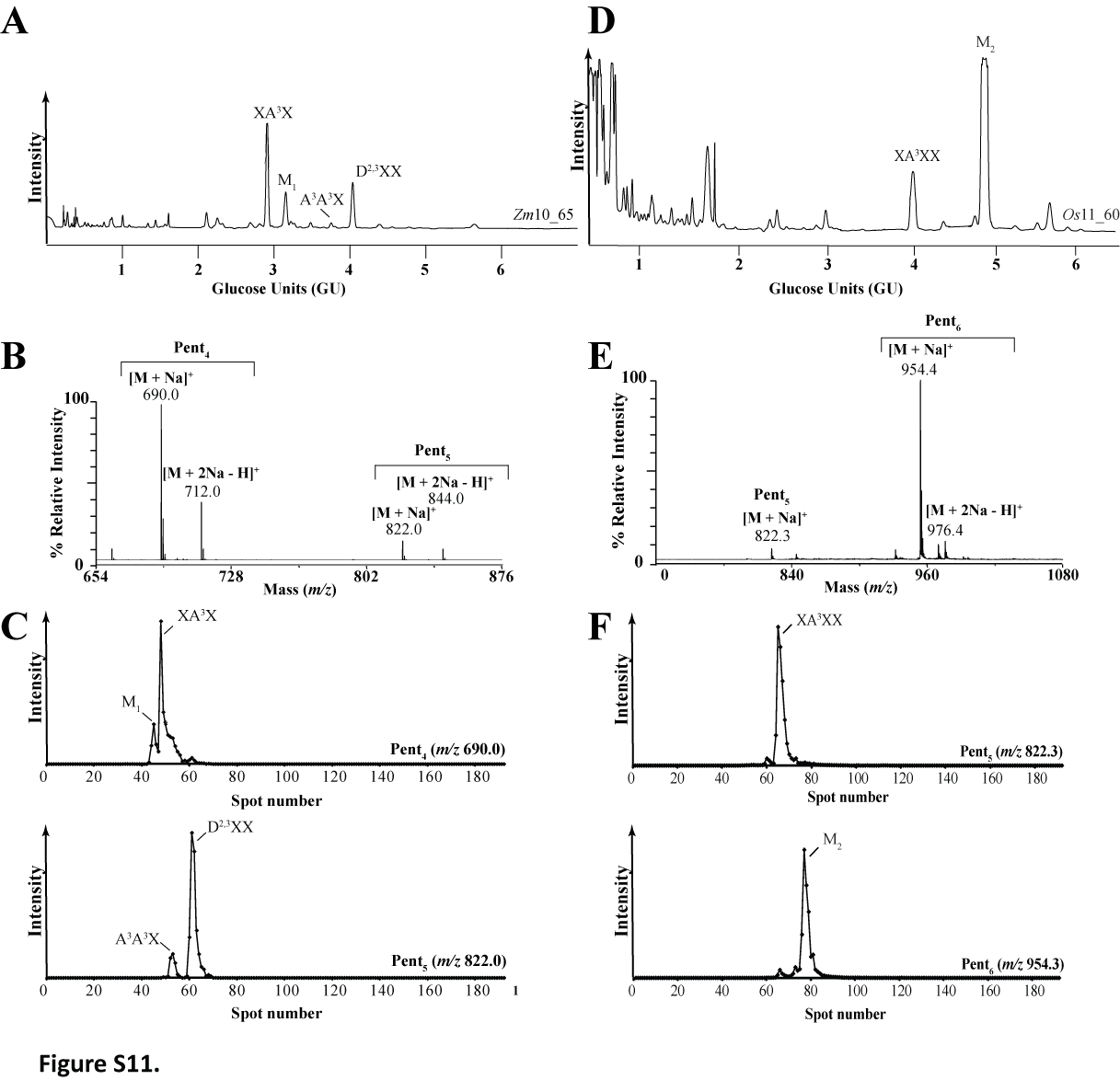


**Figure S11.** Characterisation of the oligosaccharides M_1_ and M_2._ DASH capillary electropherograms of the SEC fraction (A) *Zm*10_65 showing M_1_ peak and (B) *Os*11_60 showing M_2_ peak. Oligosaccharides characterised based on their GU are labelled. (C) MALDI-ToF-MS of fraction *Zm*10_65 showing a major sodiated and doubly sodiated ion corresponding to Pent_4_ (*m/z* 690.0 and *m/z* 712.0, respectively) and a minor sodiated and doubly sodiated ion corresponding to Pent_5_ (*m/z* 822.0 and *m/z* 844.0, respectively). (D) MALDI-ToF-MS of fraction *Os*11_60 showing a minor ion corresponding to Pent_5_ (*m/z* 822.3) and a major ion corresponding to Pent_6_ (*m/z* 954.4). (E) EIC of SEC fraction *Zm*10_65 for *m/z* 690.0 (top panel) and *m/z* 822.0 (bottom panel). (F) EIC of SEC fraction *Os*11_60 for *m/z* 822.3 (top panel) and *m/z* 954.3 (bottom panel).


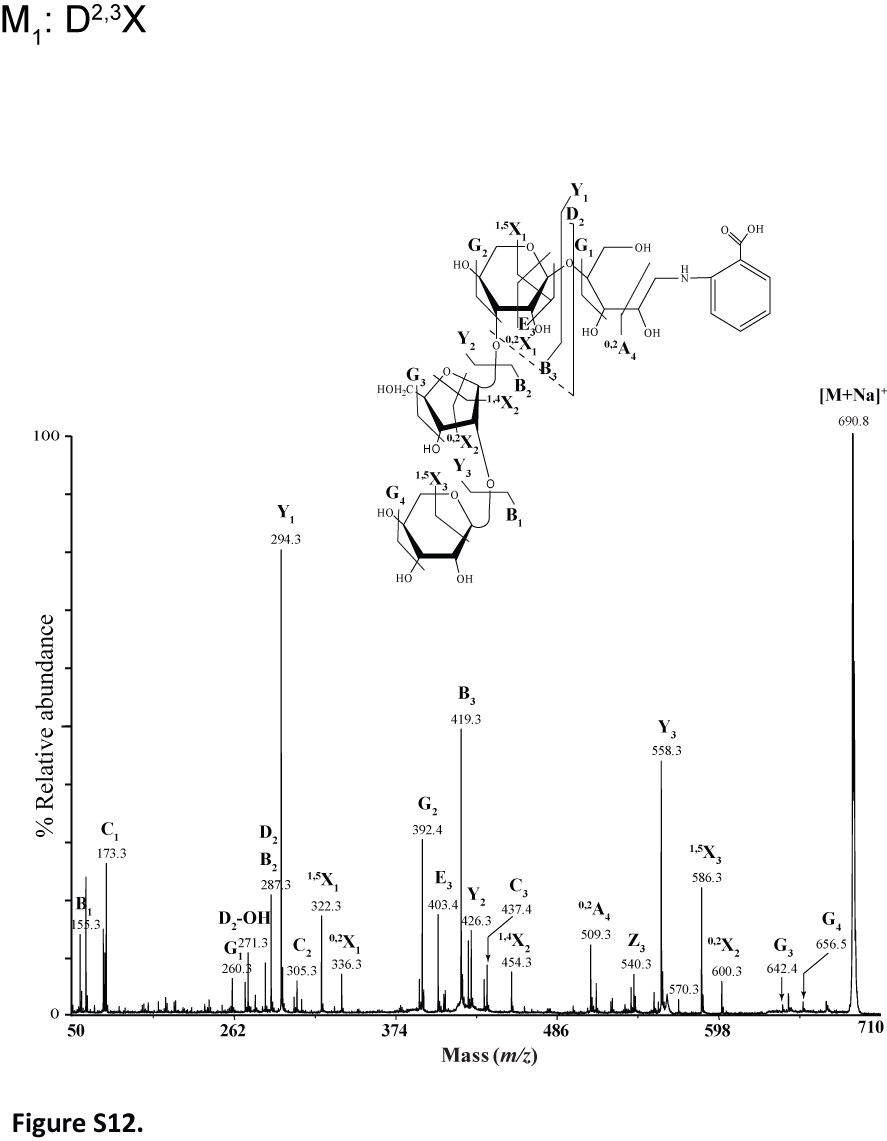


**Figure S12.** High energy MALDI-CID spectrum of the M_1_ structure: D^2,3^X.


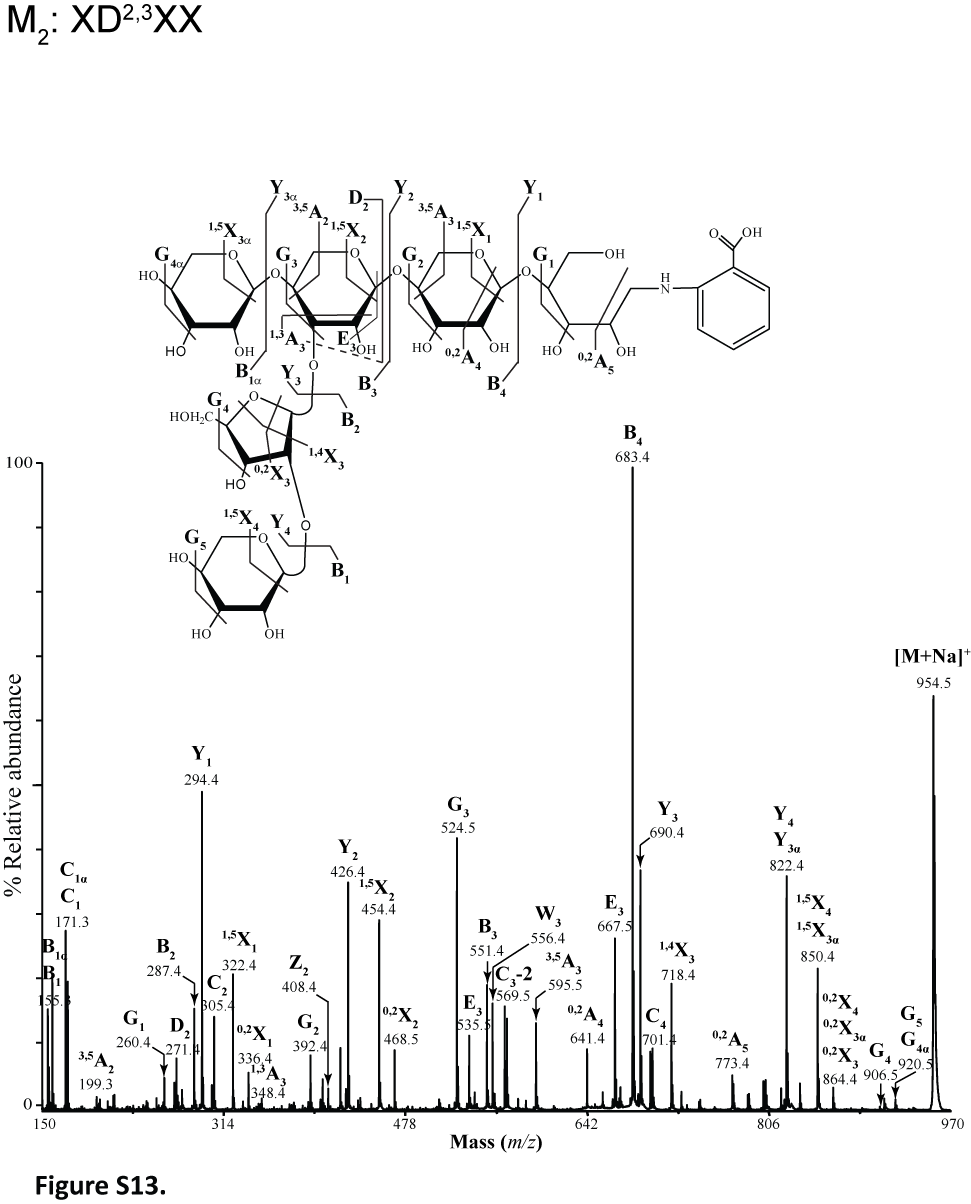


**Figure S13.** High energy MALDI-CID spectrum of the M_2_ structure: XD^2,3^XX.

Maslen, S. L., Goubet, F., Adam, A., Dupree, P., & Stephens, E. (2007). Structure elucidation of arabinoxylan isomers by normal phase HPLC-MALDI-TOF/TOF-MS/MS. *Carbohydrate Research,* 342(5), 724-735.
