# Supplementary material for: Development of an oligosaccharide library to characterise the structural variation in glucuronoarabinoxylan in the cell walls of vegetative tissues in grasses
Source: Biotechnol Biofuels. 2019 May 6;12:109. doi: 10.1186/s13068-019-1451-6 (PMC6501314; doi:10.1186/s13068-019-1451-6)
Supplement: Supplementary file 2 — Additional file 2: Table S1. 1H and 13C NMR assignments of β-Xylp-(1 → 2)-α-Araf-(1 → 3)-β-Xylp-(1 → 4)-β-Xylp-(1 → 4)-β-Xylp, at 25 °C in D2O. [file 13068_2019_1451_MOESM2_ESM.docx]

**Table S1**. ^1^H and ^13^C NMR assignments of β-Xyl*p*-(1→2)-α-Ara*f*-(1→3)-β-Xyl*p*-(1→4)-β-Xyl*p*-(1→4)-β-Xyl*p*, at 25 °C in D_2_O.

| Residue |  | Assignment | | | | |
| --- | --- | --- | --- | --- | --- | --- |
|  |  | 1 | 2 | 3 | 4 | 5 |
| β-Xyl*p*_nr_ | ^1^H | 4.557 | 3.279 | 3.448 | 3.616 | 3.971, 3.324 |
|  | ^13^C | 103.53 | 73.68 | 76.33 | 69.97 | 66.02 |
| α-Ara*f* | ^1^H | 5.510 | 4.277 | 4.112 | 4.186 | 3.824, 3.722 |
|  | ^13^C | 107.49 | 90.06 | 76.17 | 84.30 | 61.69 |
| β-Xyl*p* | ^1^H | 4.483 | 3.403 | 3.587 | 3.683 | 3.337, 3.989 |
|  | ^13^C | 102.48 | 73.74 | 82.00 | 68.56 | 65.89 |
| β-Xyl*p* | ^1^H | 4.582 | 3.375 | 3.549 | 3.780 | 3.376, 4.058 |
|  | ^13^C | n.d. | 73.59 | 74.60 | 77.20 | 63.77 |
| β-Xyl*p*_re_ | ^1^H | n.d. | n.d. | n.d. | n.d. | n.d. |
|  | ^13^C | n.d. | n.d. | n.d. | n.d. | n.d. |
